# Targeting macrophage polarization: mechanisms and potential of mesenchymal stromal/stem cells therapy in acne treatment— a narrative review

**DOI:** 10.3389/fimmu.2026.1889795

**Published:** 2026-08-12

**Authors:** Yibing Yang, Nianlv Luo, Linyan Li, Yufen Zhao, Xiaoliang Xu, Hanxiao Wei, Ying Yang, Wen Yan, Tao Zhang

**Affiliations:** 1 Department of Dermatology, Affiliated Hospital of Zunyi Medical University, Zunyi, China; 2 Key Laboratory of Cell Engineering of Guizhou Province, Affiliated Hospital of Zunyi Medical University, Zunyi, China; 3 Guizhou Biomanufacturing Laboratory, Affiliated Hospital of Zunyi Medical University, Zunyi, China; 4 Department of Burns and Plastic Surgery, Affiliated Hospital of Zunyi Medical University, Zunyi, China

**Keywords:** acne vulgaris, C. acnes, cell therapy, inflammatory response, macrophage polarization, mesenchymal stromal/stem cell

## Abstract

Acne vulgaris(AV) is a clinically common skin disease. Globally, about one in ten individuals is affected by it. Current research on acne primarily focuses on the following four areas: first, the inflammatory response; second, sebum metabolism disorders; third, the colonization of Cutibacterium acnes (C. acnes); and fourth, abnormal keratinization of the pilosebaceous duct. Although the exact pathogenesis of this condition has not yet been fully elucidated, the dual polarization of macrophages play a crucial role in the onset, progression and outcome of acne. As key effector cells in the human immune system, macrophages can exhibit different phenotypes in the microenvironments of various diseases: the classical activated phenotype (M1), the alternative activated phenotype (M2), and a subset characterized by the expression of trigger receptor 2 (TREM2) in myeloid cells. Currently endorsed international acne therapy, encompassing topical agents, systemic drugs, and physical therapies, demonstrates efficacy but frequently elicits a range of adverse effects. Therefore, there is a pressing need for a new, ideal treatment approach. Extensive research on stem cells and their derivatives in recent years has shown that mesenchymal stromal/stem cells (MSCs) possess significant immunomodulatory and anti-inflammatory capabilities, particularly in terms of influencing macrophage polarization. They promote the transition of macrophages to the anti-inflammatory M2 phenotype, which may play a role in the recovery process of acne. The unique advantage of MSCs therapy is that it is not simply antibacterial or anti-inflammatory as traditional therapies; rather, it may alleviate inflammation and promote skin tissue repair by promoting polarization of M2 macrophages. At the same time, it is expected to avoid the common adverse reactions in current acne treatment methods. This paper aims to provide a narrative review on the function of macrophages in skin tissue, the core role of inflammatory responses in acne pathogenesis, the multifaceted functions of macrophages in acne, the mechanisms through which MSCs regulate macrophage polarization, the current guideline-recommended standard treatment protocols for acne, and the feasibility of MSCs and their derivative transplants as a therapeutic strategy for alleviating acne inflammation.

## 1 Introduction

Acne vulgaris is a common chronic inflammatory dermatological condition that affects the hair follicle-sebaceous gland unit, influencing roughly one in ten individuals globally and ranking as the eighth most widespread disease worldwide (1, 2). Globally, the age-standardized prevalence of acne among adolescents and young adults aged 10-24 years increased from 8563.4 cases per 100,000 people in 1990 to 9790.5 cases per 100,000 people in 2021, with an annual increase of 0.43. The burden of acne among adolescents and adults continues to increase in almost all countries (3). Acne predominantly impacts the cheeks and forehead, subsequently affecting the chest and back, frequently resulting in a symmetrical distribution. It is often associated with enlarged pores and excessive sebum secretion. Clinical symptoms include several acne-like lesions, including comedones, papules, pustules, nodules, cysts, and scars (4). Although inflammatory erythema and persistent scarring resulting from severe acne are not uncommon in clinical practice, they can profoundly affect the physical and psychological well-being of adolescents. This may result in psychological problems, including reduced self-identity, social anxiety, and depressive mood, thereby heightening the risk of emotional and mental diseases. In severe instances, it may even contribute to suicidal inclinations (5, 6). Consequently, acne has emerged as a pressing social concern that necessitates a resolution. Current guidelines advocate for acne treatments encompassing topical drugs (such as retinoids, benzoyl peroxide (BPO), azelaic acid, antibiotic ointments), oral medications (such as antibiotics, isotretinoin, corticosteroids, and antiandrogens), and physical therapies. Although current acne treatment protocols are effective, they often provoke adverse reactions, including skin irritation, dryness, and disruption of the skin microbiota by topical medications, along with teratogenic consequences. Concerns have been raised about the rise of antibiotic-resistant microorganisms (7–10). Therefore, novel, ideal therapeutics that address the fundamental pathophysiology of acne while minimizing local and systemic adverse effects are urgently needed. As acne treatment grows more challenging, investigating new targets and mechanisms has become a pressing issue in current acne research.

The pathogenesis of acne is not fully understood, although it is mostly linked to factors such as genetics, androgen-induced overproduction of sebum, keratinization of the sebaceous duct, proliferation of C. acnes, and immune-inflammatory reactions. The immunoinflammatory response remains continuous throughout the full course of acne formation (11). Macrophages are essential to the inflammatory response and serve as pivotal constituents of the immune system, significantly contributing to the maintenance of skin homeostasis. Given the central role of inflammation in the pathogenesis of acne vulgaris, macrophages also play a significant role in the development, progression, and outcome of this condition (12, 13). Macrophages demonstrate anti-acne properties through the regulation of lipid metabolism, secretion of anti-inflammatory substances, and phagocytosis; nevertheless, their excessive activation may facilitate inflammatory responses and scar formation, hence exacerbating disease progression (14).

In recent years, the concept of “smoldering inflammation” has garnered increasing attention. Smoldering inflammation manifests as a low-intensity but persistent pathological state that is typically lacking the clinical manifestations of acute inflammation. However, at the tissue and cellular levels, it leads to persistent inflammatory infiltration. Research on this inflammation has primarily focused on multiple sclerosis (15–18), cardiovascular disease (19, 20), metabolic disorders, autoimmune diseases, cancer, and allergic diseases, serving as a key driver of the progressive deterioration of these conditions (21). Acne is essentially a chronic inflammatory skin disease characterized by chronicity, persistence, and recurrent flare-ups. This aligns closely with the concept of “smoldering inflammation.” This persistent, low-intensity inflammatory response that is difficult to resolve may be a key factor in the recurrence and chronicity of acne.

In recent years, stem cell-based therapies have achieved promising outcomes in the treatment of various conditions, including graft versus host disease (22), autoimmune disorders (23, 24), osteonecrosis of the femoral head (25), spinal cord injury (26), Parkinson’s disease (27), and skin wound healing (28). MSCs, due to their multipotent differentiation capacity, immunomodulatory characteristics, and tissue repair capacities, have significant potential for the treatment of inflammatory and autoimmune skin disorders, such as psoriasis, atopic dermatitis, contact dermatitis, alopecia areata, systemic sclerosis, and systemic lupus erythematosus, and others (29–35). Particularly, their capacity to modulate macrophages may offer a novel therapeutic approach for acne in the future. Therefore, investigating the role of macrophages in the onset, progression, and outcome of acne and exploring the potential of MSCs and their derivatives to treat acne by regulating macrophage polarization holds both theoretical and practical significance. This narrative review first introduces the functions of macrophages in the skin tissue, with a focus on the pivotal role of the inflammatory reactions in the etiology of acne. Subsequently, we reactions the intricate relationships between lipid metabolism, the colonization of C. acnes, the inflammatory response, scar formation, and macrophages. Additionally, we systematically summarize the mechanisms by which MSCs regulate the polarization of macrophages. Finally, we systematically review the current guideline-recommended acne therapy regimens, innovative treatment strategies based on macrophage polarization, and investigate the feasibility of MSCs and their derivatives transplantation as innovative therapeutic approaches, aiming to provide new insights into the pathogenesis and treatment of acne.

## 2 The function of macrophages in cutaneous tissue

### 2.1 Classification, origin, and function of cutaneous macrophages

The macrophages in the dermis are categorized into two types: tissue-resident and infiltrating. Tissue-resident macrophages predominantly derive from the yolk sac and fetal liver during embryogenesis and sustain their population postnatally via local proliferation. They are extensively distributed across the epidermis, dermis, and surrounding hair follicles, with notably high concentrations near blood vessels and nerves in the dermis (36). Infiltrating macrophages originate from monocytes, which are derived from hematopoietic stem cells in the bone marrow, and migrate to the skin solely under inflammatory or injurious conditions. They predominantly aggregate at locations of inflammation or injury, including acne lesions, eczema, or wounds (37, 38).

The tissue-resident macrophages in the epidermis are predominantly Langerhans cells (LCs), which originate from yolk sac progenitor cells and fetal liver monocytes. These cells migrate to the epidermis during embryonic development and sustain their number through self-renewal under homeostatic conditions. Circulating monocytes move to the epidermis to replace LCs populations solely under inflammation or pathological conditions. LCs, as essential elements of the skin immune system, primarily function in immunological surveillance and antigen presentation. These actions are essential for sustaining skin barrier integrity, regulating immunological homeostasis, and inhibiting viral dissemination (39, 40). Furthermore, numerous foam-like macrophages have been observed in acne lesions, and these cells have been found to express TREM2. Tran H Do demonstrated the specific overexpression of TREM2 in macrophages within acne lesions using single-cell and spatial RNA sequencing technologies, along with ultra-high-resolution Seq-Scope analysis of early acne lesions on the back skin (41). TREM2+ macrophages cannot be simply classified into the traditional M1 or M2 macrophage subsets. They represent a subset of macrophages that emerge in specific disease microenvironments and exhibit unique metabolic and functional characteristics. In acne lesions, macrophages upregulate the expression of TREM2-related genes following the phagocytosis of excess squalene, thereby differentiating into TREM2+ macrophages (41). Although TREM2+ macrophages cannot be simply classified into the M1 or M2 macrophage subsets, Tran H Do et al. found through RNA velocity analysis and pseudotime analysis that part of the TREM2+ macrophages in acne lesions differentiate from M2-like macrophages and partially overlap with M2 macrophages on the polarized continuum. This suggests that TREM2+ macrophages in acne are not a completely distinct new subtype from M2 macrophages, but rather disease-specific macrophages that overlay strong lipid metabolism and pro-inflammatory characteristics onto the M2 macrophage phenotype. Furthermore, the study also noted that there is no clear differentiation pathway between TREM2 macrophages and M1-like macrophages (41).

Tissue-resident macrophages surveil the skin microenvironment to detect and eradicate infections and aberrant cells. Under steady-state conditions, these macrophages generally display an M2 phenotype, performing anti-inflammatory and tissue-repair functions to sustain skin homeostasis. However, in response to inflammatory or damage stimuli, these cell can transition to M1 phenotype to engage in inflammatory responses (42). Tissue-resident and infiltrating macrophages collaborate through mutual coordination and functional complementarity to maintain homeostasis in the skin tissue environment. Collectively, they maintain skin immunological homeostasis, protect against pathogen incursion, and facilitate tissue regeneration.

### 2.2 Polarization of macrophages

The remarkable plasticity of macrophages is essential for their function in the skin. Macrophage plasticity refers to the ability of macrophages to dynamically adjust their phenotype and function in response to changes in their microenvironment (such as different cytokines, pathogen signals, metabolites, and activated lymphocytes) (43, 44). The process by which macrophages activate different signaling pathways in response to specific environmental stimuli and thereby differentiate into different subsets is known as macrophage polarization, which demonstrates the plasticity of macrophages. Macrophages may typically be polarized into two distinct types: classically activated M1 macrophages and alternately activated M2 macrophages (45). Lipopolysaccharide (LPS), interferon-γ (IFN-γ), granulocyte-macrophage colony-stimulating factor (GM-CSF), and tumor necrosis factor (TNF-α) typically mediate the classical activation pathway, polarizing macrophages toward the M1 phenotype. This occurs through the production of high levels of proinflammatory factors (interleukin (IL)-1β, IL-12, IL-23, iNOS, TNF-α, monocyte chemotactic protein-1, and cytotoxic mediators [reactive oxygen species (ROS) and reactive nitrogen species(RNS)], thereby augmenting inflammation and eradicating pathogens (46, 47). M2 activation occurs in response to stimulation of IL-4, IL-10, and IL-13. M2 macrophages, characterized as anti-inflammatory and reparative cells, are vital for suppressing inflammatory responses, enhancing tissue repair, and facilitating wound healing. Conversely, M2 macrophages predominantly manifest their anti-inflammatory properties via the upregulation of anti-inflammatory mediators, including IL-10, IL-6, prostaglandin E2 (PGE2), tumor necrosis factor-stimulated gene 6 (TSG-6), interleukin receptor antagonist (IL-RA), indoleamine 2,3-dioxygenase, and nitric oxide (48, 49). Their function in facilitating tissue repair and wound healing predominantly depends on the secretion of several growth factors, such as transforming growth factor-β (TGF-β), platelet-derived growth factor, and vascular endothelial growth factor (VEGF). These factors stimulate fibroblast proliferation, enhance collagen production, and facilitate neovascularization, hence expediting tissue regeneration and repair processes (50, 51). By contrast, M1 macrophages inhibit fibrosis by degrading collagen through high expression of matrix metalloproteinases (MMPs) (52).

**Figure 1:**
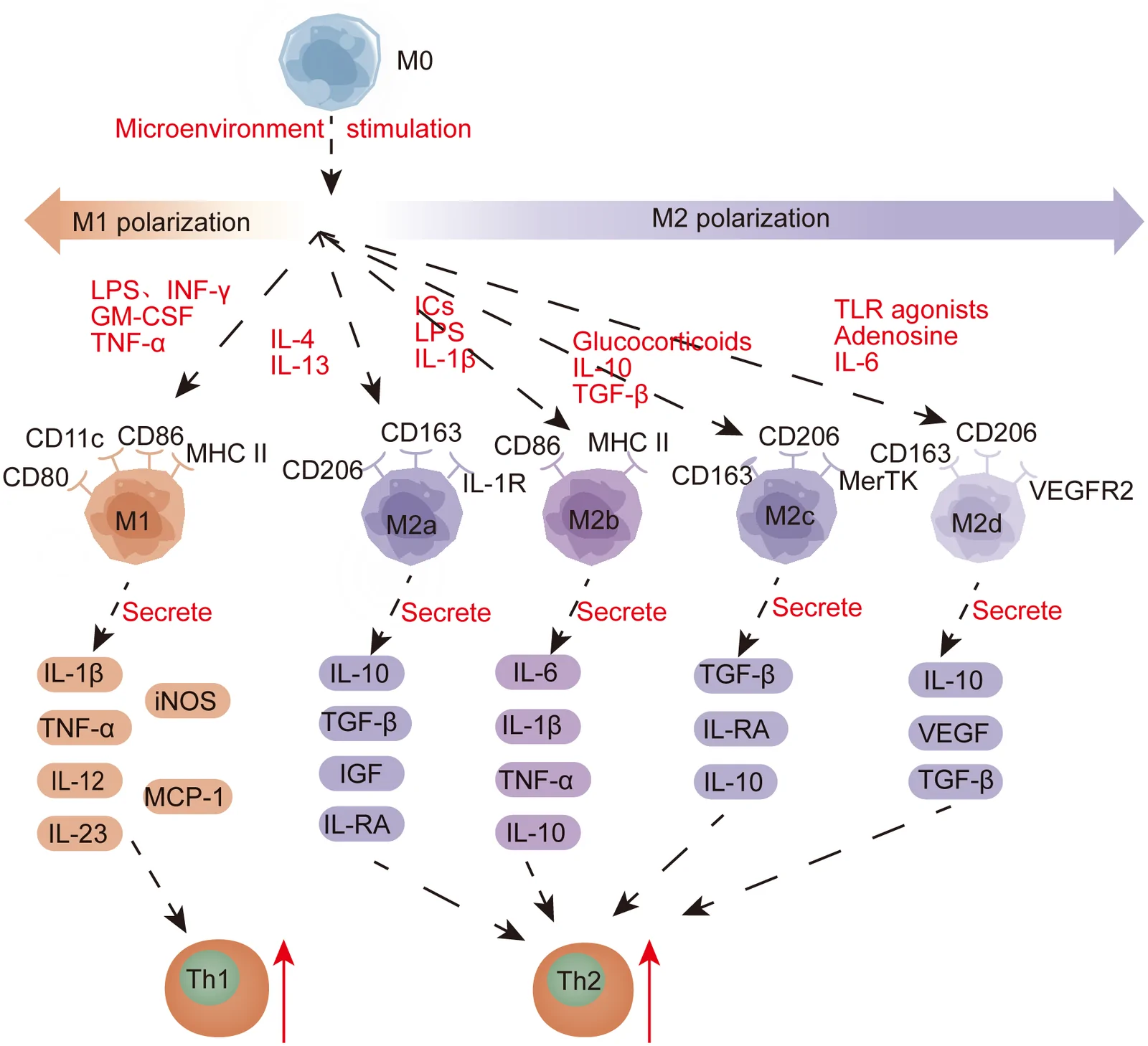
This figure shows the polarization of macrophages. Macrophages can differentiate into M1 and four M2 phenotypes under different microenvironmental stimuli. M1 predominantly performs host defense, antibacterial, and anticancer functions via proinflammatory actions; however, excessive activation may hasten the progression of chronic inflammation or autoimmune disorders. M2a is integral to anti-inflammatory responses, tissue repair, wound healing, antiparasitism, and fibrosis; M2b facilitates infection and tumor progression through the modulation of immuno-inflammatory responses; M2c exhibits significant anti-inflammatory and immunosuppressive effects while clearing apoptotic cells, although their overactivation may lead to immune evasion in chronic inflammation or autoimmune disorders; M2d is essential in the tumor microenvironment, promoting angiogenesis, tumor growth, and metastasis. M1 promotes Th1 differentiation, while all four M2 subtypes promote Th2 differentiation.

The rapid advancement of single-cell sequencing technology has facilitated a deeper exploration of macrophage heterogeneity and plasticity, challenging the traditional view that macrophages can be simply classified into M1 and M2 subtypes (53–57). Increasing evidence suggests that M2 macrophages encompass a diverse range of macrophages with significant biochemical and physiological differences (46, 55, 57–63). The simplistic classification of all such cells as M2 macrophages is overly broad; it obscures the heterogeneity among different types of M2 macrophages and fails to meet the standards of precision medicine (38, 57). Thus, M2 can be further categorized into four specific subtypes: M2a, M2b, M2c, and M2d. These subtypes vary in Inducing factors, surface indicators, secreted cytokines, and biological roles. M2a, also called wound-healing macrophages, differentiate following stimulation by IL-4 and IL-13. They are characterized by high expression of CD206, CD163, and IL-1R and secrete IL-10, TGF-β, insulin-like growth factor (IGF), and IL-RA, thereby exerting anti-inflammatory, tissue repair, wound healing, antiparasitic, and pro-fibrotic effects. M2b, also called regulatory macrophages, differentiate following stimulation by immune complexes (ICs), LPS, and IL-1β. They are characterized by high expression of CD86 and major histocompatibility complex (MHC)-II and secrete IL-6, IL-1β, TNF-α, and IL-10, thereby promoting infection and tumor progression by modulating immune and inflammatory responses. M2c, also known as inactivated macrophages, differentiate following induction by IL-10, glucocorticoids, and TGF-β. They are characterized by high expression of CD206, CD163, and MerTK, and secrete TGF-β, IL-RA, and IL-10, thereby performing functions such as phagocytosis of apoptotic cells, anti-inflammation, and immunosuppression. M2d, also called tumor-associated macrophages, differentiate following stimulation by Toll-like receptors (TLR) agonists, adenosine, and IL-6. They are characterized by high expression of CD206, CD163, and VEGFR2 and secrete IL-10, VEGF, and TGF-β, thereby promoting angiogenesis, tumor growth, and metastasis (46, 55, 59–61, 63). (**Figure 1**) summarizes the distinct biological characteristics of M1 and these four M2. Among these four M2 subtypes, the ones we often refer to as being polarized by IL-4/13 are M2a macrophages. Additionally, David M. Mosser proposed a novel classification framework based on the three fundamental functions of macrophages—host defense, wound healing, and immune regulation—analogous to the three primary colors (38).

Cytokines in the microenvironment of macrophages undergo dynamic changes, enabling flexible switching between the M1 and M2 phenotypes. This mechanism is key to maintaining the homeostasis of the immune system (49). Once this balance is disrupted, it can trigger pathological reactions and lead the body into a state of disease. M1 macrophages primarily initiate inflammatory responses and eliminate pathogens in the skin microenvironment; however, their excessive or prolonged activation may result in persistent local inflammation, leading to a chronic disease course. For example, studies have shown that in acne lesions, the sustained activation of M1 macrophages is closely associated with the long-term maintenance of inflammation. This inflammatory state can persist for months or even years, leading to a protracted and persistent course of acne (64). Given that MSCs possess potent anti-inflammatory and immunomodulatory properties. they are capable of inducing M2 polarization in macrophages and exerting anti-inflammatory effects. Thus, therapeutic approaches targeting the inflammatory response in acne, particularly which involving stem cell-mediated modulation of macrophage polarization, holds promise as a major future research direction for the treatment of acne.

## 3 The core role of inflammatory responses in the pathogenesis of acne

According to the traditional view, C. acnes colonizes the ducts of sebaceous follicles, triggering innate immune response that leads to the progression from non-inflammatory comedones to inflammatory papules, pustules, nodules, and cysts (65). However, recent studies have challenged this view. This research suggests that inflammatory responses are present at all stages of acne development and begin to play a role even during the subclinical phase, prior to the formation of comedones (66, 67). This finding challenges traditional theories regarding the pathogenesis of acne, suggesting that inflammatory responses play a central role in the disease’s pathogenesis and such responses are present throughout the entire course of the disease.

The pathogenesis of acne has not yet been fully elucidated, and the timing of the relationship between follicular infundibular keratinization and inflammatory events has long been a subject of debate. Jeremy et al. (66) analyzed biopsy specimens of clinically normal hair follicles and early-stage lesions from unaffected skin of acne patients using immunohistochemistry. They found that around hair follicles that had not yet exhibited the histological features of microcomedones, there was a significant increase in the infiltration of CD3+ T cells, CD4+ T cells, and CD68+ macrophages, while IL-1α expression was also markedly increased, suggesting that subclinical inflammation may precede the formation of microcomedones. This demonstrates that an inflammatory reaction is already present before the clinical symptoms of acne appear and may serve as an early driving factor in disease onset. In addition, Manfredini et al. (68) used reflectance confocal microscopy and dynamic optical coherence tomography to conduct a 6-week longitudinal follow-up of the same skin area in patients with mild-to-moderate acne. They observed that 1 to 2 weeks before the appearance of inflammatory papules, dilated, bright follicles (i.e., microcomedones or pre-microcomedone lesions) first appeared in the corresponding follicular infundibulum, followed by dermal inflammatory cell infiltration and enhanced vascularity in the dermal papillae. This indicates that keratinization abnormalities in the follicular infundibulum are a necessary prerequisite for triggering clinically apparent inflammation (papules, pustules). These seemingly contradictory conclusions actually reflect the multilevel nature of acne pathogenesis: the subclinical inflammation mentioned in the former refers to immune-inflammatory activation at the cellular and molecular levels, which precedes the formation of microcomedones, whereas the inflammatory events mentioned in the latter refer to clinically apparent inflammation (such as visible papules and pustules), for which microcomedones are a necessary prerequisite. In short, immune-inflammatory activation at the cellular and molecular levels drives the formation of microcomedones, which then further amplify the inflammatory reaction. The above conclusion is mainly based on studies with small samples and short time periods. In the future, larger-scale and longer-term longitudinal studies are still needed to further clarify the exact temporal relationship between the two.

The pathological process of acne begins with the formation of microcomedones, a process closely linked to two aspects: first, excessive sebum secretion; and second, abnormal proliferation and hyperkeratinization of keratinocytes in the infundibulum of the hair follicle (69). Excessive keratinization of keratinocytes can block the hair follicle duct, preventing the normal discharge of sebum and ultimately leading to the formation of microcomedones. As sebum continues to accumulate, the follicular structure progressively enlarges while keratin debris persistently accumulates, propelling microcomedones to progress into clinically observable comedones. This series of changes constitutes the initial stage of acne onset. Comedones are classified into two types: whiteheads (closed comedones) and blackheads (open comedones). The former is characterized by a completely closed pore beneath the epidermis. This prevents the sebum plug from being exposed to air, so it appears white or close to the natural skin tone. The latter is caused by partial closure at the opening of the hair follicle duct, causing the sebum plug to oxidize when exposed to air (70). These blocked hair follicles and accumulated sebum provide an anaerobic environment and nutrients for C. acnes, leading to its rapid proliferation and triggering an inflammatory response, which clinically manifests as localized redness and swelling of the skin. If untreated, the skin lesions may progress to papules, pustules, nodules, and cysts, and may even result in stubborn scarring, causing severe psychological distress to the patient (14, 71).

**Figure 2:**
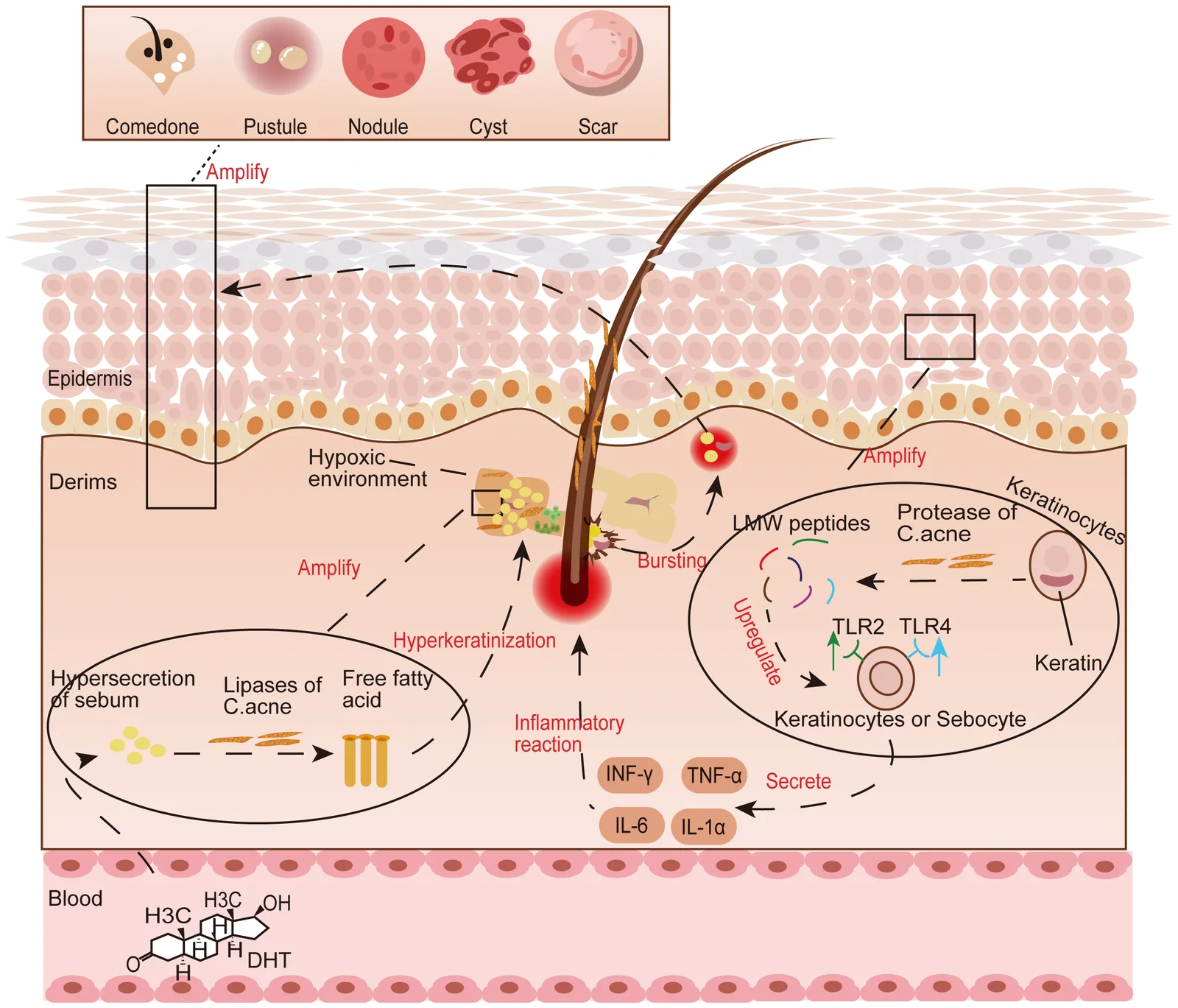
This figure shows the four core pathogenesis mechanisms of acne, including androgen-induced hypersecretion of lipids from the sebaceous glands, abnormal keratinization of the follicular sebaceous duct, proliferation of C. acnes, and immune inflammatory responses.

The internationally acknowledged pathogenesis of acne is the result of the combined action of four core pathogenic factors (72, 73). Androgen metabolism disorders can lead to excessive sebum secretion, accompanied by abnormal proliferation and keratinization of the hair follicle epithelium. This condition provides the nutrients and anaerobic environment required for the overgrowth of C. acnes. Subsequently, low-molecular-weight peptides—metabolic byproducts of C. acnes—induce the production of pro-inflammatory factors in the follicle and surrounding tissues, triggering an inflammatory response. This prompts immune cells to migrate and infiltrate the follicular wall. This chronic inflammatory process may ultimately rupture the follicular wall, allowing contents to spill into the dermis. This results in clinical manifestations including papules, pustules, nodules, cysts, and scarring (**Figure 2**).

Extensive research indicates that C. acnes infiltrating the sebaceous glands of hair follicles stimulates the secretion of multiple bioactive enzymes, including lipases, proteases and hyaluronidases. These enzymes may disrupt the follicular wall, resulting in widespread inflammation, foreign body granuloma formation, and scarring (74). Among these proteins, lipases and proteases are crucial to the advancement of acne. Lipases decompose triglycerides in sebum into free fatty acids, stimulating the proliferation and hyperkeratinization of keratinocytes in the follicular duct (75). The latter can decompose structural proteins (such as keratin) and extracellular matrix components (such as collagen) within keratinocytes into low-molecular-weight peptides. These low-molecular-weight peptides can act as antigens or inflammatory mediators that are identified by the host immune system. They stimulate pattern recognition receptors (PRRs), including TLRs, on keratinocytes and sebaceous gland cells, thereby increasing the expression of TLR2 and TLR4. This then enhances the production of IL-1α, IL-6, TNF-α, and INF-γ, initiating a series of downstream reactions (76). This process involves the activation of two signaling pathways, NF-κB and MAPK. This activation induces the polarization of macrophages toward the M1 phenotype and promotes the release of pro-inflammatory cytokines (77, 78). The continued intensification of the inflammatory response can cause the hair follicle wall to rupture, allowing the contents of the follicle, including keratin and hair, to enter the dermis. These contents will be recognized as foreign substances by the host’s immune system, triggering inflammation around the hair follicle sebaceous gland unit and ultimately leading to a range of clinical manifestations, from papules to cystic lesions. Additionally, IL-1β and TNF-α can significantly upregulate the expression of matrix metalloproteinases (including MMP-1, MMP-3, and MMP-9) by activating the MAPK signaling pathway. Among these, the upregulation of MMP-9 is the most pronounced (78, 79). These proteases disrupt the normal structure of skin tissue by degrading collagen fibers in the extracellular matrix. During the healing process of post-acne erythema, this process leads to disruption of the local dermal structure and imbalance between collagen synthesis and degradation, thereby setting the foundation for pathological scar formation (76).

The chronic, protracted, and recurrent features of acne are closely associated with “smoldering inflammation” (80). This inflammation manifests as a low-intensity but persistent pathological state, typically lacking the clinical manifestations of acute inflammation. However, at the tissue and cellular levels, it leads to persistent inflammatory infiltration. As a transitional state between acute inflammation and complete remission, smoldering inflammation plays a role in various chronic diseases, including metabolic disorders, autoimmune diseases, cardiovascular diseases, and skin conditions (such as acne and psoriasis) (21). In acne lesions, chronic, low-grade inflammation may persist long after clinical symptoms have subsided. This smoldering inflammation is a major factor in acne recurrence or disease progression. This makes acne management more challenging, requiring a dual-pronged approach: first, controlling visible inflammation; and second, addressing smoldering inflammation.

Overall, the inflammatory response triggered by C. acnes is a core driver of the onset, progression, and chronicity of acne. It persists throughout all stages of the disease. From the activation of the innate immune response to persistent smoldering inflammation, this inflammatory response not only drives disease progression but also leads to tissue remodeling and scar formation. Consequently, the targeted suppression of C. acnes-induced inflammation has become a pivotal therapeutic strategy in acne management.

## 4 The multiple roles of macrophages in acne

Macrophages play a dual role in the progression of acne: on one hand, they serve as key effector cells in immune surveillance; on the other hand, they act as initiators of the inflammatory response. Its primary functions include regulating lipid metabolism, phagocytosing C. acnes, and mediating the inflammatory response. However, when the immune response is overactivated, macrophages may trigger excessive inflammatory cascades, leading to tissue damage or scar formation. Clarifying the complex biological behavior of macrophages in acne is crucial for maintaining skin immune homeostasis and optimizing treatment strategies.

### 4.1 Macrophages and lipid metabolism

In recent years, studies have suggested a potential underlying association between acne and metabolic disorders. Certain experts assert that acne is essentially a metabolic disorder, and have validated it through cross-sectional studies (81, 82). Further study revealed a strong correlation between metabolic syndrome and the onset of acne. The incidence of metabolic abnormalities is markedly elevated among acne patients, often accompanied by typical metabolic disorders such as increased body mass index, impaired glucose metabolism, abnormal blood pressure, and dyslipidemia (83). Dysregulation of lipid metabolism is a central pathological feature of acne and is considered a key precondition for its development. The main components of sebum include squalene, triglycerides, free fatty acids, wax esters, and cholesterol (82). Excessive sebum secretion can induce abnormal keratinization of the hair follicle ducts and disrupt the balance of the microbial community. Additionally, it can trigger immune dysregulation and a cascade of inflammatory reactions, ultimately leading to the formation of characteristic skin lesions (84).

Studies have shown that, compared to healthy individuals, the lipid profiles of acne patients exhibit distinct abnormalities, specifically manifested as markedly elevated levels of total serum cholesterol, triglycerides, and low-density lipoprotein (LDL) cholesterol, while high-density lipoprotein cholesterol levels are markedly reduced (83, 85). This disruption of lipid profiles is particularly prominent in patients with severe acne, suggesting a possible correlation between abnormal lipid metabolism and the severity of acne (86).

In the context of the aforementioned lipid metabolism disorders, macrophages act as key regulatory cells involved in the regulation of LDL and cholesterol. Macrophages express the macrophage scavenger receptor 1 (MSR1) and TLRs. Through these receptors, macrophages phagocytose excess sebum produced by the sebaceous glands as well as oxidatively modified lipids (such as oxidized LDL), leading to the accumulation of lipids within the cells (85, 87). Studies have shown that prolonged hypoxia induces the polarization of macrophages toward the pro-inflammatory M1 phenotype by activating the Akt and β-catenin signaling pathways. In the early stages of acne, excessive sebum secretion blocks the sebaceous follicle opening, thereby creating a hypoxic environment. Under hypoxic conditions, macrophages are promoted to polarize toward the M1 phenotype, which stimulates the release of IL-1β and TNF-α, thereby enhancing the local inflammatory response (88). Furthermore, studies have shown that macrophages that have phagocytosed excessive lipids (particularly squalene) transform into foam cells and express TREM2. In acne lesions, TREM2+ macrophages are a subset of cells with a unique expression profile of lipid metabolism and inflammation-related genes. Their capacity to metabolize lipids and produce pro-inflammatory factors is markedly enhanced. The unique double-bond structure of squalene also endows it with a highly efficient ability to scavenge ROS. This not only depletes the ROS essential for the antibacterial activity macrophages but also suppresses the induction of oxidase enzymes. Ultimately, this impairs the antibacterial function of macrophages, preventing them from effectively reducing C. acnes load. This discovery provides a theoretical foundation for the mechanism of action of topical BPO in treating acne. This mechanism involves the generation of ROS, which counteract the antioxidant effects of squalene. This mechanism involves the generation of ROS, which counteract the antioxidant effects of squalene. Specifically, squalene inhibits macrophage antimicrobial activity by scavenging oxygen-free radicals, while ROS generated by BPO effectively neutralize this inhibitory effect, thereby restoring and maintaining macrophage antimicrobial function. Moreover, squalene stimulates TREM2+ macrophages to release proinflammatory cytokines and chemokines (such as IL-18, IL-1β, CXCL9 and CCL5) while inhibiting the synthesis of the anti-inflammatory factor IL-10, leading to intensified local inflammatory responses (41). A study by Min Deng et al. further elucidated the mechanism by which TREM2+ macrophages drive inflammatory responses (89). When the granulin precursor secreted by TREM2+ macrophages in acne lesions binds to sortilin 1 on their surface, it activates the downstream NF-κB signaling pathway, leading to the production and release of large amounts of pro-inflammatory cytokines and chemokines. In addition to impairing bactericidal function and driving inflammatory responses, MMPs secreted by TREM2+ macrophages also exacerbate follicular wall rupture and abnormal tissue remodeling (41, 89).

Macrophages perform multiple roles in the metabolism in lipid of acne. Dysfunction of macrophages is closely associated with the progression of acne, as these cells serve as a bridge between lipid metabolism and inflammatory responses. A deeper understanding of the specific role macrophages play in the lipid metabolism of acne is crucial for the development of new therapeutic strategies. Intervention strategies may focus on the following areas: enhancing cholesterol efflux, blocking the formation of foam cells, and promoting the polarization of macrophages toward the M2 phenotype. In addition, TREM2+ macrophages exhibit pathogenic characteristics in acne. Although there is currently no direct evidence reporting the modulatory effects of MSCs on TREM2+ macrophages in acne lesions, previous studies have documented the regulatory role of MSCs on TREM2+ macrophages in the context of other diseases (such as Alzheimer’s disease and pulmonary fibrosis) (90, 91). This suggests that an MSCs intervention strategy targeting TREM2+ macrophages may become one of the effective treatments for acne in the future.

### 4.2 Macrophages and C. acnes

C. acnes, formerly known as Propionibacterium acnes, is a Gram-positive, anaerobic rod-shaped bacterium. It is a component of the normal human skin flora. This bacterium primarily colonizes areas rich in sebaceous glands, such as the face, chest, and back, and is closely associated with the development and progression of acne (92). During the pathogenesis of acne, C. acnes plays multiple pathogenic roles. Endogenous porphyrins produced during its metabolism promote the oxidation of squalene, which then induces abnormal proliferation and differentiation of keratinocytes, ultimately leading to the formation of typical acne lesions (93).

Sorel Fitz-Gibbon and colleagues used metagenomic and whole-genome sequencing technologies for the first time to reveal differences in the skin microbiome between acne patients and healthy individuals at the strain level. The research found that the C. acnes strains on the skin of acne patients were primarily composed of RT4, RT5, and RT7–10 strains, whereas those of healthy individuals were dominated by RT1, RT2, and RT6 strains. This study provided the first evidence that the pathogenicity of C. acnes is strain-specific. Furthermore, the team proposed that a disruption in the dynamic balance between “pathogenicity” and “commensalism” may be the core mechanism driving the development of acne. This finding provides a theoretical foundation for future therapies targeting pathogenic strains of C. acnes and enhances the precision of acne management (94).

During the host immune defense process, macrophages recognize pathogen-associated molecular patterns (PAMPs) via PRRs, initiate phagocytosis, and trigger an inflammatory response, thereby playing a central role in killing C. acnes (95). Research indicates that when C. acnes interacts with macrophages, both its cell wall components and metabolic byproducts act as PAMPs. These substances stimulate TLR2 on the macrophage membrane, thereby enhancing the expression levels of IL-15 and GM-CSF through the NF-κB signaling pathway. Subsequently, macrophages rapidly differentiate into two cell types: IL-15-dependent CD209+ macrophages and GM-CSF-dependent CD1b+ dendritic cells (96, 97). Philip T. Liu and his colleagues found that these two cell subsets perform different functions. CD209-positive cells exhibit a macrophage-like phenotype, and their phagocytic efficiency is twice the rate of CD1b+ cells. CD1b+ cells exhibit an immature dendritic cell phenotype and act as professional antigen-presenting cells for T lymphocytes. Although CD1b+ cells have weak direct antibacterial capabilities, they can activate Th1-type adaptive immune responses by secreting IL-12 and TNF-α (96, 98). CD1b+ cells eliminate pathogens through adaptive immune responses, but they may also exacerbate chronic inflammation. This research team further discovered that all-trans retinoic acid (ATRA) plays a dual role in regulating macrophage differentiation: on one hand, ATRA directly promotes the differentiation of CD209+ macrophages; on the other hand, when stimulated by C. acnes or IL-15, ATRA further enhances the differentiation of CD209+ macrophages. This will enable a 1%–25% increase in the proportion of CD209-positive cells. Notably, ATRA simultaneously inhibits GM-CSF-mediated CD1b+ cell differentiation. This implies that ATRA can both upregulate CD209-positive cells differentiation and downregulate pro-inflammatory CD1b+ cells, thereby exerting both antibacterial and anti-inflammatory effects. These findings provide an important molecular theoretical basis for the future use of ATRA in the treatment of acne (98).

### 4.3 Macrophage-driven inflammatory responses in the progression of acne

Due to their heterogeneity and significant plasticity, macrophages can alter their phenotype and function in response to various microenvironmental stimuli, such as infection, tissue damage, metabolic abnormalities, or T-cell responses (99). During the course of acne, macrophages polarize into M1 or M2 types in response to different stimuli at various stages (46). This plasticity is crucial in processes such as chronic inflammation, cancer, and maintaining physiological homeostasis (100, 101). Research demonstrates that macrophages next to sebaceous glands in healthy skin are primarily of the M2 phenotype, while M1 macrophages are largely observed seen in acne lesions (42, 43). Besides, research has demonstrated that in acne lesions, excessive activation of the pro-inflammatory M1 or insufficient function of the anti-inflammatory M2 leads to an imbalance in the M1/M2 ratio. This imbalance exacerbates local inflammatory responses and promotes inflammatory damage to the hair follicle-sebaceous gland unit (102, 103). In the initial phases of acne formation, C. acnes proliferates significantly in the anaerobic conditions of the follicular sebaceous duct. Macrophages identify the PAMPs of C. acnes via their TLRs, initiating polarization toward the M1 phenotype. This process exacerbates local inflammation at the lesion site and eliminates C. acnes by producing proinflammatory factors and cytotoxic mediators such as ROS and RON (95). The inflammatory cytokine IL-1β is crucial in acne-related inflammation. Research has demonstrated that IL-1β expression levels are positively correlated with the clinical severity of acne and the extent of histopathological inflammation (104). Recent research by Tang et al. Revealed the crucial role of the sebaceous gland microenvironment in regulating macrophage phenotypes. Experimental data indicate that, under inflammatory conditions associated with acne, sebaceous glands can promote the polarization of macrophages toward the M1 phenotype. Notably, using a specialized *in vitro* acne model they established, the research team demonstrated for the first time the multifaceted anti-acne effects of cannabidiol (CBD): first, it significantly improves abnormal lipid metabolism; second, it alleviates oxidative stress; third, it downregulates the expression levels of pro-inflammatory factors; and fourth, it reverses the abnormal polarization process of macrophages. These findings provide an important theoretical basis for the future application of CBD in acne treatment (105). In addition, Lovászi and his team further elucidated how lipids secreted by sebaceous glands regulate macrophage polarization and function. Palmitic acid, stearic acid, and oleic acid act synergistically with C. acnes to activate the TLR2/4 pathway, thereby increasing the secretion of IL-1β and exacerbating the local inflammatory response. Conversely, linoleic acid alleviates local acne inflammation through the suppression of pro-inflammatory factors such as IL-1β, IL-6, and TNF-α. Lovászi et al. further found that macrophages treated with oleic acid or linoleic acid significantly upregulated M2 markers (including CD206, CD209, and FXIII-A). It is worth noting that a contradiction appears to exist here: oleic acid simultaneously exhibits both pro-inflammatory effects (manifested in enhanced IL-1β secretion) and anti-inflammatory effects (manifested in the promotion of M2 macrophage polarization) (106).

As acne gradually transitions from the acute inflammatory phase to the clinical remission phase, both clinical symptoms and the intensity of inflammation diminish. However, the condition has not yet reached a state of complete resolution; rather, it evolves into a chronic inflammatory state characterized by low-intensity but persistent inflammation. During this transitional phase, the distribution of macrophage subsets at the lesion site changes, specifically with a gradual decrease in M1 macrophages and a gradual increase in M2 macrophages (42, 80). M2 macrophages exert their anti-inflammatory effects by downregulating the production of pro-inflammatory factors while simultaneously upregulating the secretion of anti-inflammatory factors. Additionally, M2 macrophages release various growth factors, thereby promoting tissue regeneration and wound healing (107).

### 4.4 The impact of macrophages on scar formation

M2 macrophages typically exhibit beneficial biological effects in various pathological conditions. However, overactivation of these cells can lead to excessive accumulation of TGF-β. TGF-β exerts several effects: first, it promotes fibroblast proliferation and differentiation into myofibroblasts; second, it inhibits the activity of matrix metalloproteinases; and third, it reduces the degradation of collagen and elastic fibers. Collectively, these effects lead to increased collagen deposition, ultimately resulting in scar formation (108, 109). Thus, an imbalance in M1 and M2 macrophages directly influences the pathological progression of scar formation (110). Scars that form in the later stages of acne can generally be divided into two categories: atrophic scars and hypertrophic scars. Bussabong Chancheewa et al. reported high densities of myofibroblasts in hypertrophic acne scars, while atrophic scar specimens exhibited no regions abundant in myofibroblasts. Consequently, they posited that the elevated myofibroblast density in hypertrophic scars is associated with prolonged macrophage activation (111). Recent research by Julei Zhang et al. demonstrated that sonic hedgehog (SHH) accelerates skin scar progression by augmenting the oxidative phosphorylation pathways in macrophages, therefore promoting M2 polarization (112). Consequently, combined therapeutic approaches targeting macrophage metabolism and polarization phenotypes may achieve optimized scar repair outcomes (such as the use of vismodegib to inhibit SHH signaling).

M1 predominantly contributes to atrophic scars, unlike hypertrophic scars. Studies have demonstrated that in the early inflammatory phase of acne, aberrant activation of M1 results in the overproduction of pro-inflammatory mediators, including IL-1β, IL-6, and TNF-α, thus triggering scar formation. These inflammatory substances not only intensify local inflammatory responses surrounding hair follicle but also increase the production of MMPs. This results in the destruction of type I and III collagen while concurrently suppressing fibroblast synthesis of new collagen, leading to deficiencies in the dermal matrix. This ultimately results in skin depressions, which clinically manifest as boxcar scars, rolling scars, and pitted scars (111, 113).

**Figure 3:**
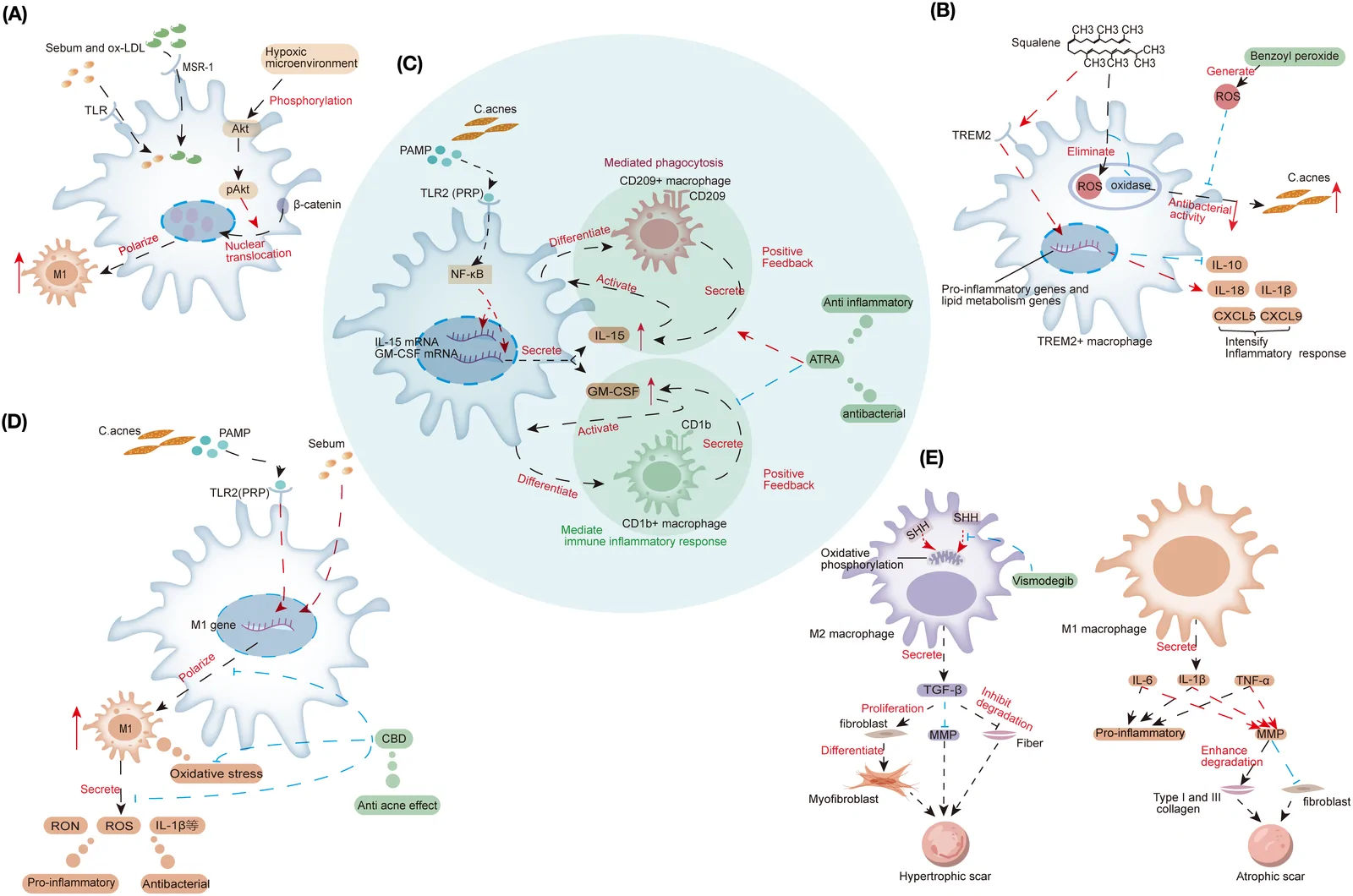
This figure shows the multifaceted role of macrophages in acne. **(A, B)** macrophages and lipids; **(C)** macrophages and C. acnes; **(D)** macrophages and inflammation; **(E)** macrophages and scars (including hypertrophic scars and atrophic scars). The red dotted arrows indicate “upregulation/promotion”; the blue dotted blunt-ended lines represent “downregulation/inhibition”. **(C)** is the section within the light blue circle.

Xuechuan Li et al. performed a case-control study demonstrating that keloids displayed significantly elevated M2 levels and markedly higher NR3C1(glucocorticoid receptor) expression than healthy controls did, with a positive association between the two variables. This work emphasizes the function of M2 in facilitating scar formation and partially elucidates the individual variability noted in clinical glucocorticoid therapy (114). Recent investigations has revealed that SPP1+ macrophages with M2 phenotype, along with POSTN+ fibroblasts, facilitate keloid development via SPP1-axis-mediated interactions. Clinically, localized corticosteroid injections directed at this axis markedly improve disease activity (115) (**Figure 3**).

### 4.5 Macrophage function during different stages of acne: a preliminary model

Different polarization states of macrophages exert complex bidirectional regulatory effects at various stages of acne progression (14). In the early stages of acne, M1 macrophages are activated by recognizing PAMPs on the surface of C. acnes and participate in the clearance of C. acnes. However, the inflammatory reaction triggered by the excessive activation of M1 macrophages accelerates the progression of acne. Although M2 macrophages possess anti-inflammatory and tissue-repair capabilities, they are also key drivers of scar formation in the later stages of acne (108, 109). This suggests that macrophage function is not static but rather changes dynamically as the disease progresses. A study by Hong et al. observed that as acne keloidalis progresses from the subacute to the chronic phase, macrophages gradually shift from the M1 to the M2 phenotype (115). Currently, however, no literature systematically elucidates the specific subtype distribution and functions of macrophages across the various stages of acne, and our understanding of the precise spatiotemporal patterns of macrophages in acne remains limited. Therefore, we can currently only construct a dynamic regulatory model with stage-specificity and functional plasticity as a theoretical hypothesis. In this model, the course of acne is divided into three stages: the early stage, the inflammatory infiltration stage, and the resolution and repair stage. In the early stage of acne, partial macrophages polarize into the M1 phenotype by recognizing PAMPs on the surface of C. acnes and eliminate C. acnes by releasing pro-inflammatory factors and cytotoxic mediators. Upon entering the inflammatory infiltration phase, M1 macrophages are extensively activated in a high C. acnes load environment, further enhancing their bactericidal capacity. Once the bacterial load falls below a certain threshold, M1 macrophages rapidly shift toward the M2 phenotype, limiting the spread of inflammation. During the resolution and repair phase, M2 macrophages play a role in tissue remodeling. However, appropriate interventions are required at this stage to prevent their sustained activation from leading to scar formation. This model is still in its preliminary stages and will need to be validated using multidimensional techniques in the future. If this model can be successfully established in the future, it may provide a theoretical basis for the staged and precise intervention of acne.

## 5 MSCs modulate macrophage polarization

MSCs originate from the mesoderm and represent a type of adult stem cell with multipotent differentiation capabilities. They can differentiate into cells such as osteoblasts, chondrocytes, and adipocytes and are widely distributed in tissues including bone marrow, adipose tissue, peripheral blood, dental pulp, muscle, amniotic membrane, umbilical cord, and placenta (116). MSCs have emerged as a central research focus in tissue engineering and regenerative medicine because to their unique biological characteristics, including accessible tissue sources, exceptional *in vitro* and *in vivo* proliferation capacity, potent immuno-inflammatory regulatory properties, multilineage differentiation potential and superior tissue repair capabilities. These characteristics of MSCs offer them broad prospects in both basic research and clinical applications, particularly in the treatment of inflammatory and autoimmune skin diseases. Furthermore, MSCs exhibit low expression of MHC class I molecules on their surfaces and rarely or almost never express MHC class II molecules. This unique molecular expression confers a distinct immunological property of low immunogenicity, endowing MSCs with significant immune privilege. During allogeneic transplantation, they evade immune recognition, thereby facilitating tissue repair. These findings provide a theoretical basis for MSC transplantation therapy in inflammatory and autoimmune skin diseases (117, 118).

In 2006, the International Society for Cellular Therapy (ISCT) proposed minimum criteria for defining human MSCs: “MSCs must exhibit plastic adhesion under standard culture conditions; and MSCs must express CD105, CD73, and CD90, and not express CD45, CD34, CD14, or CD11b, CD79a, CD19, and HLA-DR surface molecules; MSCs must differentiate *in vitro* into osteoblasts, adipocytes, and chondrocytes (119). As fundamental research and clinical trials involving MSCs are gradually being conducted, notable differences in experimental results and treatment effectiveness have been identified among cells that meet the criteria mentioned above. The previous version of the standard, established in 2006, was based solely on surface markers and differentiation potential and is no longer sufficient to meet the needs of current research (120). In 2019, ISCT updated its definition criteria for MSCs, highlighting that “mesenchymal stem cells” are not equivalent to “mesenchymal stromal cells.” The former emphasizes “stemness,” particularly self-renewal and multipotent differentiation capabilities, whereas the latter predominantly focus on secretory, immunomodulatory, and homing properties. The abbreviation “MSC” is still utilized; however, owing to the heterogeneity of cells from different tissue sources, the specific tissue origin must be included (e.g., BM-MSCs, AD-MSCs, UC-MSCs, PB-MSCs, etc.) (121). Although MSCs from various tissue sources possess the fundamental biological characteristics of MSCs, those derived from different tissues exhibit distinct tissue specificities. For instance, BM-MSCs were the first discovered MSC, with the most extensive research, abundant clinical safety data, and strong osteogenic potential. Nonetheless, the invasive collection method may result in supply constraints (122, 123). A remarkable feature of AD-MSCs is the simple acquisition of adipose tissue, rich MSC content, relatively low cost, absence of immune rejection in autologous transplantation (appropriate for autologous use), and lack of tumorigenic potential (124, 125). UC-MSCs demonstrate superior proliferation potential and can be sourced from discarded umbilical cords, avoiding ethical controversies. They have remarkably low immunogenicity and tumorigenicity, rendering them appropriate for autologous transplantation (126). In recent years, PB-MSCs have emerged as a hotspot in regenerative medicine research owing to their advantages of easy and non-invasive collection and their appropriateness for autologous transplantation. The naturally low abundance of MSCs in peripheral blood complicates the acquisition of adequate cell quantities for fundamental experiments or clinical research. A recent study indicated that pretreatment with granulocyte colony-stimulating factor (G-CSF) greatly improved the recovery rate of peripheral blood MSCs. Zhang et al. effectively separated MSCs from mobilized rat peripheral blood and revealed that G-CSF mobilization significantly increased the number of MSCs from peripheral blood, with the colony formation rate increasing by approximately 60-fold, thereby obtaining the necessary scale for experimentation (127). MSCs derived from alternative tissues have distinct advantages and disadvantages. The selection in basic research and clinical trials should be predicated on a thorough assessment of illness type, cell acquisition difficulties, proliferation capability, differentiation potential, and treatment goals.

Early research on MSCs was limited by a focus on their “stem cell-like properties”, attributing therapeutic benefits to the cells’ directed chemotaxis, proliferation, and differentiation capabilities. However, as studies progressed, it became evident that despite issues such as low post-transplantation survival, low retention rates, and low differentiation rates observed in numerous clinical trials, MSCs still demonstrated favorable therapeutic effects (128, 129). The true therapeutic effect of MSCs in various disease microenvironments stems from their immuno-inflammatory regulatory capacity rather than simple cell replacement effects (130, 131).

**Figure 4:**
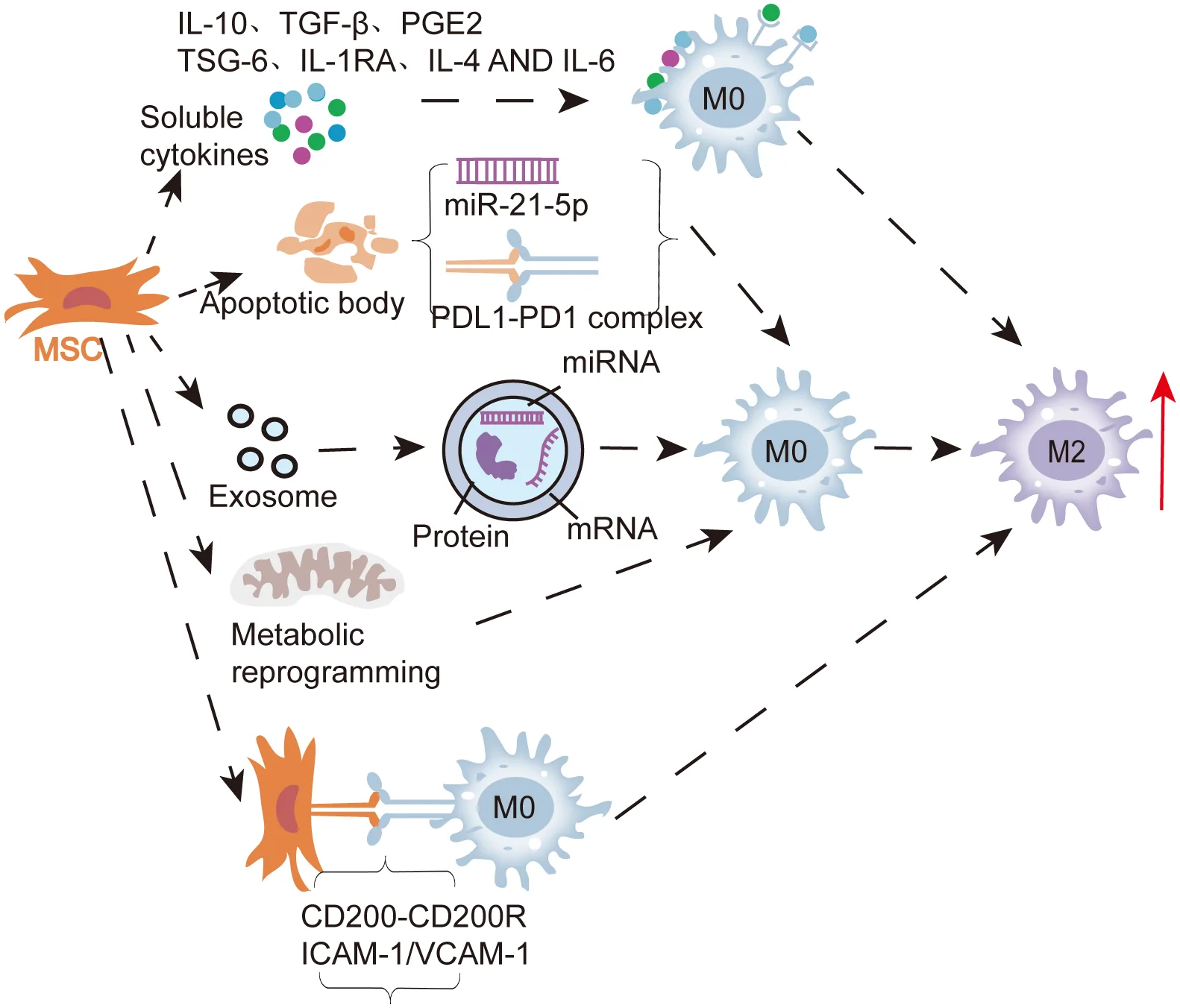
This figure shows the mechanisms by which MSCs regulate macrophage polarization. Specific mechanisms include paracrine effects (encompassing soluble factors, exosomes, and apoptotic bodies), metabolic reprogramming, direct cell-to-cell contact and so on.

However, the mechanisms by which MSCs regulate macrophage polarization remain incompletely understood. Therefore, gaining deeper insights into the mechanisms involved in MSC-mediated macrophage polarization could facilitate the development of novel therapeutic strategies for acne. The following primarily outlines the currently well-established regulatory mechanisms and signaling pathways (**Figure 4**).

### 5.1 MSCs regulate macrophage polarization through paracrine effects

One of the most widely recognized mechanisms is that MSCs regulate macrophage plasticity through paracrine effects (132). In the early stages of MSC research, which were limited by the cognitive level at the time, it was believed that MSCs achieved therapeutic effects by migrating to the injury site, leveraging their multidirectional differentiation potential and proliferative characteristics to differentiate into target cell types and replace damaged tissue cells through quantitative expansion. However, numerous clinical trials have demonstrated that a major obstacle to effective MSC therapy is the inability to efficiently deliver these cells to target tissues (129). Therefore, the paracrine effect may be the main mechanism by which MSCs exert their therapeutic effects.

MSCs can modulate macrophage polarization through the secretion of various soluble factors (including IL-10, PGE2, and TGF-β) and extracellular vesicles (EVs) such as exosomes, microvesicles (MVs), and apoptotic bodies (ABs), thereby increasing anti-inflammatory M2 differentiation and suppressing pro-inflammatory M1 activation (132–134).

#### 5.1.1 MSCs regulate macrophage polarization by secreting soluble factors

IL-10 is a critically important anti-inflammatory cytokine, and the anti-inflammatory response it mediates is essential in numerous inflammatory disorders (135). Moreover, research has shown that serum IL-10 levels are markedly diminished in individuals with acne, underscoring the vital role of IL-10 in the development of this condition (136). Zhang et al. illustrated *in vitro* through a Transwell co-culture system that PB-MSCs activate the JAK1/STAT3 pathway in macrophages via the secretion of IL-10, consequently facilitating M2 polarization and suppressing the release of proinflammatory cytokines such as TNF-α and IL-1β, thereby inhibiting inflammatory responses (137). Furthermore, the team further verified through animal experiments that PB-MSCs can induce M2 macrophage polarization *in vivo* via the IL-10/STAT3 signaling pathway, thereby correcting the excessive inflammatory response in a rat acne model and promoting the healing of acne lesions (138, 139). Similarly, studies have shown that both BM-MSCs and cervical cancer-derived MSCs can induce the polarization of M2 macrophages by secreting IL-10 (140, 141).

Prostaglandin E2 (PGE2), the primary end product of the cyclooxygenase-2 metabolic pathway, plays a crucial role in the immunomodulatory function of MSCs (142, 143). An *in vitro* study demonstrated that macrophages co-cultured with BM-MSCs significantly suppressed the secretion of TNF-α and IL-6, while enhancing the expression of IL-10, compared to macrophages cultured alone. Further mechanistic studies indicate that this cytokine shift is closely associated with the phenotypic transformation of macrophages, and that macrophage polarization during this process is regulated by PGE2 (143). Subsequent investigations into molecular mechanisms demonstrated that PGE2 interacts with the EP2 and EP4 receptors on macrophages, thereby activating adenylate cyclase and elevating cAMP levels. This results in the activation of protein kinase A (namely the cAMP/PKA pathway) and the subsequent phosphorylation of cAMP response element-binding protein (CREB) (Serr113), hence augmenting C/EBP-β transcription. C/EBP-β additionally enhances the expression of the anti-inflammatory cytokine IL-10 and the M2 marker Arg1 (144). Additionally, a study has shown that TGF-β can facilitate the promotion of M2 macrophages via the Smad2/3 pathway (145).

Similarly, the TGF-β produced by MSCs plays a vital role in modulating macrophage plasticity. Liu et al. reported that TGF-β released by MSCs enhances M2-like polarization of LPS-stimulated macrophages via increased phosphorylation of Akt (Ser473) and FoxO1 (Thr24) in *in vitro* co-culture experiments. Facilitating the polarization of LPS-stimulated macrophages toward the M2 phenotype inhibits the release of pro-inflammatory factors and augments phagocytic activity (146). Additionally, other soluble factors released by MSCs, such as TSG-6 (147), IL-1RA (148), IL-4 (149), and IL-6 (150), participate in regulating macrophage plasticity, thereby exerting a significant regulatory effect on the inflammatory microenvironment and immune responses.

#### 5.1.2 MSCs regulate macrophage polarization by secreting EVs

MSC-derived EVs (MSC-EVs) is another important mechanism for regulating macrophage polarization. Traditionally, EVs can be classified into three distinct subtypes based on their size and biogenesis: exosomes (diameter range of 50–150 nm), MVs (diameter range of 100–1,000 nm), and ABs (diameter range of 50–4,000 nm) (151). Exosome exhibit the most exceptional biological properties among all EV types, making it the primary focus of current EV research (131).

MSC-EVs are rich in various bioactive molecules, including non-coding RNAs, messenger RNAs, and proteins. These bioactive molecules mediate the immunomodulatory functions of MSCs. Furthermore, they can be taken up by macrophages via endocytosis, thereby regulating macrophage polarization (152). MicroRNAs, a category of non-coding RNA, constitute one of the most significant bioactive molecules transported by EVs. They operate within the MSC/macrophage axis, regulating macrophage polarization (153). For example, Zhao et al. revealed that MSC-Exo facilitate M1-to-M2 polarization by transferring miR-182, which suppresses the TLR4/NF-κB pathway and stimulates the PI3K/Akt pathway in macrophages (154). Additionally, Huang et al. and Ji et al. experimentally established that exosomes from BM-MSCs and ectopic endometrial stromal cells (ESCs) can facilitate the polarization of macrophages (microglia) toward the M2 phenotype by delivering miR-146a-5p, which targets tumor necrosis factor receptor associated-factor 6 (TRAF6) (155, 156). Furthermore, other microRNAs contained in MSC-Exos, including miR-233 (157), miR-6733 (158), and miR-24 (159), also participate in regulating macrophage phenotypic conversion.

MSC-Exos not only retain nearly all the biological properties of MSCs but also exhibit greater stability, effectively avoiding numerous limitations inherent in traditional MSC transplantation therapies. These limitations manifest in several ways: first, low MSC survival rates following implantation; second, low homing and retention rates in target organs; and third, the potential risk of tumorigenesis (131). In addition, MSC-Exos are capable of crossing the blood-brain barrier and entering the central nervous system (160).

#### 5.1.3 MSCs regulate macrophage polarization by secreting apoptotic bodies

Apoptosis refers to programmed cell death, which is irreplaceable for the development, growth, and immunological functions of organisms (161). During apoptosis, cells experience a sequence of morphological alterations that can produce many forms of EVs, including exosomes, microvesicles, and apoptotic bodies, with the latter being the primary product of apoptotic cells. ABs are the most substantial type of extracellular vesicle and are abundant in DNA, RNA, proteins, and organelles (162).

We noted that despite lower survival rates and durations following transplantation, the anti-inflammatory and immunomodulatory effects mediated by MSCs endure (163, 164). Specifically, apoptotic MSCs even exhibit stronger immunomodulatory properties (165). Thus, we hypothesize that ABs formed from apoptotic MSCs (MSC-ABs) may play a crucial role in sustaining this long-term effect. Due to their sustained anti-inflammatory and immunomodulatory effects, MSC-ABs hold significant potential for the intervention and treatment of chronic inflammatory skin diseases (such as acne). Liu et al. experimentally established that BM-MSC-ABs can induce macrophages to polarize toward the M2 phenotype, hence facilitating anti-inflammatory and tissue healing capabilities. This ultimately expedites wound closure, diminishes scar formation, and enhances collagen deposition and re-epithelialization (164). Nevertheless, Liu et al. did not conduct additional research on the specific active factors and downstream signaling pathways by which BM-MSC-ABs influence macrophage polarization. Conversely, Li et al. identified the molecular mechanism by which AD-MSC-ABs facilitate skin wound repair: miR-21-5p which the ABs possess targets and suppresses KLF6 mRNA expression, thereby activating M2 polarization and ultimately expediting skin wound healing (166). In addition, Jiang and his team discovered another mechanism by which MSC-ABs regulate macrophage polarization: after transplantation, MSCs undergo apoptosis at the site of inflammation, releasing ABs that express programmed cell death 1 ligand 1 (PD-L1) on their surface. When phagocytosed by alveolar macrophages, the ABs bind to programmed cell death protein 1 (PD-1) on the macrophage surface. The PD-L1–PD-1 interaction inhibits Erk1/2 phosphorylation, which then downregulates the gene expression of three key rate-limiting enzymes in glycolysis (Hk3, Pgam, and Ldha), shifts macrophage metabolism toward oxidative phosphorylation, and ultimately promotes M2 macrophage polarization (163). Meanwhile, Su et al. developed a fibrous scaffold capable of carrying BM-MSC-ABs. This scaffold locally releases ABs at the wound site; after being phagocytosed by macrophages, the ABs deliver mmu-miR-21a-5p, which directly targets and inhibits the CCL-1 gene, thereby promoting M2 macrophage polarization (167). In addition to accelerating skin wound healing, MSC-ABs have demonstrated substantial therapeutic potential in multiple clinical conditions. Liao et al. found, utilizing a rat model of intrauterine adhesions, that MSC-ABs can promote M2 macrophage polarization. This process reduces the release of IL-1β and TNF-α and increases the expression of IL-10 and VEGFA (168). In addition, a study led by Zheng has further enriched the mechanism by which apoptotic bodies regulate macrophage polarization. After apoptosis, MSCs release apoptotic vesicles that expose calreticulin on their surface. This calreticulin mediates the phagocytosis of apoptotic vesicles by hepatic macrophages, which then induces transcriptional reprogramming of hepatic macrophages, ultimately promoting M2-type macrophage polarization (169). Wang and his team conducted a primary evaluation of the immunomodulatory capabilities of MSC-ABs of different sizes. They found that Large-ApoBDs exhibited superior immunomodulatory effects, such as T-cell suppression and macrophage polarization. This result may be attributed to the larger surface area, higher ligand density, or more efficient release of their contents (170).

In comparison to exosomes, ABs are still in the early stages of development in the field of immunomodulation. The literature reports are relatively scarce, necessitating more in-depth exploration.

### 5.2 MSCs regulate macrophage polarization through direct cell-to-cell contact

MSCs can regulate macrophage polarization through direct cell-to-cell contact. For example, MSCs express the CD200 molecule on their surface, which binds to the CD200 receptor on macrophages. This process conveys inhibitory signals to macrophages, facilitating M2 macrophage polarization and diminishing the inflammatory response of macrophages (152). Additionally, PD-L1, a crucial immunoregulatory molecule on the surface of MSCs, exerts immunomodulatory effects through its interaction with PD-1. Shalini et al. revealed that the interaction PD-L1 with PD-1 on macrophages diminishes JNK and STAT1 phosphorylation, which inhibits Elk-1/c-Fos and thus leads to substantial reductions in proinflammatory cytokines IL-12 and TNF-α, resulting in anti-inflammatory effects (171). We propose that this may entail a transition in macrophage phenotype from pro-inflammatory M1 to anti-inflammatory M2. Du et al. further demonstrated that inhibiting PD-L1 interaction with PD-1 prompts macrophages to polarize toward the M1 subtype by examining the correlation between the PD-1/PD-L1 signaling pathway and macrophage polarization in preeclampsia (172).

Moreover, MSCs can modulate macrophage polarization by expressing cell and vascular adhesion molecules (such as ICAM-1 and VCAM-1) that interact with integrins on the macrophage membrane. However, the molecular mechanisms underlying this process remain to be further elucidated. Notably, MSCs modulate macrophage plasticity by direct cell-to-cell contact, which may function as a supplementary mechanism within the local microenvironment rather than the principal channel, in contrast to the paracrine effects of MSCs. Currently, research into the mechanisms that directly regulate macrophage polarization is still in its infancy. Traditional Transwell cell culture techniques are unable to eliminate the effects of exosomes and soluble factors, which means that many fundamental mechanisms in this field remain unexplained. We urgently need more precise experimental models and methods to address these existing limitations.

### 5.3 MSCs regulate macrophage polarization through metabolic reprogramming

Metabolic reprogramming refers to the process by which cells actively alter their metabolic pathways in order to adapt to environmental changes (such as hypoxia, inflammation, or cell-cell interactions). This process modifies energy supply mechanisms, biosynthetic pathways, and signaling functions to meet the demands of the environment (173). In recent years, metabolic pathways underlying various basic cellular functions have garnered widespread attention. Among these, the polarization process of macrophages may be closely related to changes in their energy metabolism pathways (144). M1 and M2 macrophages exhibit distinct metabolic characteristics due to their different functions. M1 macrophages rely on anaerobic glycolysis, aerobic glycolysis (the Warburg effect), and the tricarboxylic acid cycle to rapidly generate sufficient energy for the secretion of pro-inflammatory factors and phagocytosis. In contrast, M2 macrophages are primarily involved in long-term tissue repair and anti-inflammatory responses, so they require a relatively sustained energy supply. Thus, mitochondrial oxidative phosphorylation and fatty acid oxidation are more appropriate for M2 macrophages (144, 173, 174). Consequently, we can boldly hypothesize that the transformation of the macrophage phenotype from M1 to M2 involves a metabolic pathway shift from aerobic/anaerobic glycolysis to oxidative phosphorylation. Jan et al. confirmed this by demonstrating that M1 macrophages encounter difficulties in differentiating into M2 due to mitochondrial dysfunction (175). Moreover, a report indicated that the M1-to-M2 polarization of macrophages during myocardial infarction is closely linked to a transition from glycolysis to the tricarboxylic acid cycle, hence corroborating this (176).

Reports suggest that MSCs modulate macrophage polarization via PGE2-dependent metabolic reprogramming, transitioning from the M1 to M2 phenotype. This process involves the suppression of glycolysis, activation of the AMPK/SIRT1 pathway, and enhancement of oxidative phosphorylation and fatty acid oxidation (177). Moreover, research has demonstrated that UC-MSCs metabolically reprogram monocytes via lactate secretion, facilitating M2 polarization and encouraging monocyte development into dendritic cells (178). Moreover, MSC-ABs can facilitate the transformation of macrophages to an anti-inflammatory phenotype by mediating their metabolic reprogramming (163).

Furthermore, recent studies have shown that MSCs can transfer mitochondria to macrophages via extracellular vesicles and tunneling nanotubes, thereby promoting M2 macrophage polarization. This process involves an increase in the level of oxidative phosphorylation in macrophages (153). A study by Morrison et al. demonstrated that MSCs can transfer mitochondria to alveolar macrophages by secreting EVs containing functional mitochondria, thereby enhancing the macrophages’ levels of oxidative phosphorylation. This process enhances the macrophages’ phagocytic capacity, promotes M2 macrophage polarization via nanotunnels, and upregulates the secretion of anti-inflammatory factors (179). Furthermore, in addition to EV-mediated transport, tunneling nanotubes represent another important pathway for MSCs to transfer mitochondria to macrophages. Since 2004, when Rustom et al. first described nanotunnels as a new mechanism of intercellular communication (180), Megan V. Jackson was the first to demonstrate *in vitro* that MSCs can transfer mitochondria to macrophages via nanotunnels. Furthermore, in a subsequent study using a mouse ADRS model, Jackson demonstrated that MSCs can transfer mitochondria to macrophages by forming tunnel nanotubes, thereby enhancing macrophage oxidative phosphorylation and, consequently, their phagocytic and anti-inflammatory capabilities (181).

The aforementioned studies demonstrate that MSCs can modulate the plasticity of macrophages by influencing metabolic pathways via many mechanisms, including soluble factors, lactate, apoptotic bodies, and mitochondrial transfer. The capacity of MSCs to modulate the immuno-inflammatory response through metabolic reprogramming pathways has been extensively investigated; nevertheless, the role of this mechanism in the management of acne requires further investigation.

### 5.4 Small summary

MSCs modulate macrophage plasticity via multiple methods, including synergistic actions of various factors, intercellular interactions, and metabolic alterations. These overlapping pathways collectively promote the transformation of macrophage phenotypes. In addition to the aforementioned classical regulatory pathways, MSCs can influence macrophage phenotypes via processes such as phagocytosis by macrophages (182) or by altering macrophage epigenetic changes, such as DNA methylation (183). MSC-mediated macrophage phenotype regulation plays a crucial role in immuno-inflammatory modulation, and its therapeutic potential has been validated in various inflammatory or immune-related diseases. Nonetheless, investigations in the domain of acne are still rather limited. Few studies have investigated the impact of MSCs on acne pathogenesis via macrophage polarization, resulting in largely unexamined therapeutic potential. The capacity of MSCs to regulate macrophage polarization and hence intervene in inflammatory responses in the acne microenvironment has been established. Strategies targeting this pathway may provide new perspectives for the development of innovative acne treatments.

## 6 Conventional treatments for acne vulgaris, therapeutic strategies based on regulating macrophage polarization, and potential therapeutic approaches involving the transplantation of MSCs and their derivatives

Currently, treatment strategies for acne require individualized intervention plans based on the severity of the patient’s condition and the type of skin lesions. For mild to moderate cases, first-line treatments typically involve topical administration, including monotherapy or combination formulations such as retinoids, BPO, and azelaic acid. Moderate-to-severe cases necessitate consideration of systemic oral medications. Common clinical regimens include tetracycline antibiotics (such as doxycycline or minocycline), antiandrogens (such as the oral contraceptive pill or spironolactone), and isotretinoin (7, 184). While current acne therapies are somewhat effective, they require prolonged treatment durations, suffer from poor patient adherence, and frequently cause a range of side effects. Topical treatments often induce adverse effects such as skin irritation, dryness, and disruption of the skin microbiota, while systemic therapies may result in more serious consequences such as hepatic and renal damage or teratogenic hazards (185). In addition, long-term use of antibiotics can lead to the emergence of super-resistant bacteria. Therefore, there is an urgent need to develop a new treatment approach that can effectively avoid the side effects mentioned above while maintaining significant therapeutic efficacy.

The pathogenesis of acne has not yet been completely clarified, but the prevailing view currently focuses on four main mechanisms: first, androgen-induced hyperkeratinization; second, hyperkeratinization of the pilosebaceous duct; third, the colonization of C. acnes; and fourth, the inflammatory response (73). Contemporary treatments for acne are based precisely on these four mechanisms. In recent years, in-depth research into the pathophysiology and immune-inflammatory responses during the progression of acne has driven an update in treatment strategies. The focus has shifted from simple antibacterial and anti-inflammatory approaches to more precise mechanisms, particularly the control of immune inflammation and the prevention of scarring (14). Considering the crucial function of macrophages in the genesis, progression, and outcome of acne, treatments targeting macrophages may offer a new approach to acne treatment. This therapy involves mitigating inflammation by inhibiting inflammatory signaling pathways and cytokine synthesis, including approaches that promote M2 polarization (14). In recent years, MSCs have been shown to possess remarkable immunomodulatory and anti-inflammatory capabilities, particularly in regulating macrophage plasticity, making them a promising treatment for inflammatory and autoimmune skin diseases. Therefore, we propose investigating the potential of transplanting MSCs or their derivatives to facilitate M2 macrophage polarization, thus mitigating the immuno-inflammatory response in acne and intervening in disease progression. This section outlines therapeutic methods for acne from three perspectives: current standard clinical therapies, novel approaches based on the polarization of macrophages, and transplantation therapies involving MSCs and their derivatives.

### 6.1 Standard clinical treatment

#### 6.1.1 Topical retinoids

Retinoids are vitamin A compounds that are esteemed in dermatology and are available for topical use and oral intake. Topical retinoids are the foundation of acne therapy, and are utilized either as a standalone treatment or in conjunction with other topical or systemic drugs (185). Topical retinoids are classified into six categories: ATRA, adapalene, tazarotene, trifarotene, alitretinoin, and bexarotene (186). The first four medicines have received approval from the U.S. Food and Drug Administration (FDA) for acne therapy (187).

The pharmacological effects of retinoids are mediated mainly by their particular binding to nuclear retinoic acid receptors (RARs). There are three RAR subtypes, RAR-α, RAR-β, and RAR-γ, with RAR-γ exhibiting the greatest expression levels in human skin (187). Based on molecular structure and receptor affinity, retinoids are classified into four generations. Among these, the fourth-generation drug trifarotene improves therapeutic efficacy while reducing drug toxicity. It exhibits extremely high affinity for RAR-γ. Notably, trifarotene is the first clinically validated topical retinoid that can be used for both trunk and facial acne (188, 189). Retinoids can simultaneously target various pathogenic mechanisms of acne. They improve abnormal keratinization in the sebaceous follicular ducts, suppress inflammatory responses, and reduce sebum secretion; however, their core mechanism lies in correcting abnormal keratinization in the pilosebaceous ducts (187). Specifically, retinoic acid normalizes skin keratinization by accelerating the turnover of hair follicle epithelial cells and the shedding of keratinocytes, thereby promoting the expulsion of mature comedones and inhibiting the formation of microcomedone (186). Therefore, topical retinoids serve as a cornerstone of acne treatment. However, treatment with retinoids is typically a long-term process and can cause skin irritation, which may lead to reduced patient compliance. Dong et al. have found that certain skincare products can effectively alleviate the side effects of retinoids through their barrier-repairing properties and anti-inflammatory ingredients, thereby effectively reducing the local side effects of topical retinoid medications (190).

#### 6.1.2 Oral retinoids

Oral retinoids, represented by isotretinoin, serve a crucial role in the treatment of acne. Isotretinoin is a core medication used to treat severe (particularly nodulocystic) or stubborn acne (185). Currently, oral isotretinoin is the only medication capable of simultaneously targeting all four major pathogenic mechanisms of acne. Its core mechanism lying in its ability to effectively inhibit sebum secretion (74, 191). Specifically, isotretinoin reduces the size of sebaceous glands and suppresses sebum production, thereby indirectly inhibiting the proliferation of C. acnes (192). In addition, oral isotretinoin possesses the ability to regulate follicular keratinization and exhibits anti-inflammatory properties (193). One of the molecular mechanisms by which isotretinoin inhibits sebum secretion is its ability to upregulate the expression levels of certain transcription factors (including p53, FoxO1, and FoxO3) in acne lesions. This triggers sebocyte death and reduces BLIMP1(+) sebaceous progenitor cells, ultimately resulting in a reduction in sebum secretion (194). This biological process also elucidates several adverse effects of isotretinoin, such as teratogenicity (apoptosis of neural crest cells), diminished ovarian reserve (apoptosis of granulosa cells), heightened risk of depression (apoptosis of hippocampal neurons), and hyperlipidemia (mediated by p53). Additionally, isotretinoin may induce undesirable effects such as xerosis, cheilitis, epistaxis, heightened skin sensitivity, and altered hepatic function (185, 195). Among these, dry skin and mucous membranes are the most common; nonetheless, teratogenicity and dyslipidemia warrant the highest level of vigilance.

Isotretinoin, as one of the core medications for acne treatment, has a broad spectrum of side effects. Nonetheless, the majority of adverse effects are dose dependent and can be efficiently controlled by preventive strategies (196). As the only medication currently capable of simultaneously targeting all four major pathogenesis mechanisms of acne, it has remarkable clinical efficacy. Therefore, under strict adherence to indications and standardized monitoring of adverse reactions, isotretinoin remains irreplaceable within the treatment regimen for refractory acne.

#### 6.1.3 BPO

Benzoyl peroxide, as a topical antimicrobial agent, is the treatment of choice for mild to moderate acne. It acts through multiple mechanisms, including eradicating C. acnes, dissolving comedones, and exerting anti-inflammatory effects (72). Specifically, the oxygen free radicals generated by BPO can replenish those eliminated by squalene. The oxygen free radicals produced by BPO can directly eliminate C. acnes (41). BPO is currently the only acne treatment proven to markedly alter skin microbial biodiversity (197). BPO can promote stratum corneum renewal and alleviate obstruction of the pilosebaceous duct (198). Studies have shown that the combination of BPO with retinoids can enhance the anti-acne effect by reducing excessive keratinization around the hair follicle and lowering keratin levels within the follicle (199). BPO also disrupts neutrophil membranes through its cytotoxic activity, thereby reducing ROS release (200). By reducing ROS release, it exerts anti-inflammatory effects. By reducing ROS release, it exerts anti-inflammatory effects. Benzoyl peroxide is not prone to inducing drug resistance and remains effective against resistant C. acnes strains. Therefore, it is often used in combination with antibiotics such as clindamycin. This therapy enhances antimicrobial efficacy while markedly reducing the emergence of drug-resistant strains (198, 201–203).

Common adverse effects of BPO include a burning sensation, stinging, dryness, erythema, pain, peeling, irritation, fabric staining, bleaching, and infrequent contact allergies. The frequency and intensity are correlated with the drug concentration (204). Interestingly, a previous study conducted the gel-sol microencapsulation of BPO. The silica coating on the drug surface functions as a physical barrier, slowing the release rate of BPO to prevent an initial surge of high-concentration drugs. This provides the skin with additional time to adapt to the drug’s irritation, effectively diminishing the irritancy of BPO (204). Research has demonstrated that 2.5%, 5%, and 10% BPO formulations have comparable duration of clinical response and efficacy; however, elevated doses may provoke irritating dermatitis. Consequently, in clinical practice frequently reduces BPO concentrations to alleviate negative effects (198).

#### 6.1.4 Azelaic acid

Azelaic acid offers multiple therapeutic benefits in the management of acne: first, it has antibacterial properties; second, it corrects abnormal keratinization; third, it has anti-inflammatory effects; fourth, it acts as an antioxidant; and fifth, it reduces hyperpigmentation (205, 206). Most physicians prescribe it as a second-line topical treatment for mild to moderate acne (205). Owing to its skin-lightening and hyperpigmentation-reducing properties, it is more appropriate for acne patients with sensitive skin or darker complexions (185), but it poses a risk of inducing hypopigmentation. Aside from hypopigmentation, the adverse effects of azelaic acid are usually modest and commonly present as skin irritation reactions (198).

Moreover, research has demonstrated that azelaic acid can suppress sebum production. Anna Szymańska’s two clinical trials, comprising 35 and 27 female participants respectively, both reported reduced sebum production, indicating that azelaic acid prolonged the inhibition of sebaceous activity (207, 208). Although the precise mechanism remains unclear, azelaic acid has potential as a future therapeutic agent targeting all four major pathogenesis mechanisms of acne.

#### 6.1.5 Antibiotics

In standard acne management guidelines, antibiotic use encompasses both topical and oral administration, targeting the two core pathways involves in acne pathogenesis: excessive proliferation of C. acnes and inflammatory responses (185, 209).

##### 6.1.5.1 Topical antibiotics

The American Academy of Dermatology (AAD) advocates the use of topical antibiotics as the first-line treatment for moderate acne (210). Three FDA-approved topical antibiotics for the treatment of acne in both children and adults include clindamycin, erythromycin, and minocycline (72). Clindamycin is the antibiotic of choice for topical acne treatment. Clindamycin is the antibiotic of choice for topical acne treatment. The mechanism of action against C. acnes involve binding to the peptidyl transferase center of the 50S ribosomal subunit, hence altering tRNA placement and peptide chain elongation, which inhibits bacterial protein synthesis (211, 212). The specific anti-inflammatory mechanism is as follows: clindamycin diminishes neutrophil chemotaxis to sites of inflammation; inhibits TLR-2 expression, thereby downregulating the release of pro-inflammatory mediators; and suppresses C. acnes, consequently reducing the production of pro-inflammatory substances such as lipases, free fatty acids, porphyrins, hyaluronidase, and matrix metalloproteinases (211). Notably, the well-documented therapeutic effectiveness of antibiotics in acne management has resulted in prolonged, excessive, or indiscriminate use, resulting in an increase in the prevalence of drug-resistant C. acnes, especially those resistant to erythromycin and clindamycin (213). Therefore, guidelines do not recommend monotherapy with topical antibiotics. Integrating them with BPO not only increases their effectiveness but also effectively inhibits the emergence of antibiotic resistance (185).

##### 6.1.5.2 Oral antibiotics

Indications for systemic oral antibiotics include moderate to severe acne, acne resistant to previous topical treatments, or extensive acne lesions (209). A variety of antibiotics can be used for the treatment of acne, including penicillins (amoxicillin and ampicillin), macrolides (erythromycin, roxithromycin, and azithromycin), lincosamides (clindamycin), fluoroquinolones (levofloxacin), and tetracyclines (doxycycline, minocycline, and tigecycline), among others (208, 209). Among these, tetracyclines are extensively used for acne treatment owing to their minimal resistance rates (185), high liposolubility, and cost-effectiveness (209), constituting 75% of oral antibiotic prescriptions in dermatology (214). Tetracycline suppresses protein synthesis in C. acnes by binding to the 30S ribosomal subunit, effectively eradicating the bacteria (215). Its anti-inflammatory mechanism specifically involves the suppression of neutrophil chemotaxis, the downregulation of pro-inflammatory molecules including IL-1β, IL-8, and TNF-α and the inhibition of metalloproteinases (215). In additionally to resistance, tetracycline can induce various side effects, such as photosensitivity, dysbiosis, gastrointestinal distress, inflammatory bowel disease, hyperpigmentation, lupus erythematosus, idiopathic intracranial hypertension, pharyngitis, dizziness, and vertigo (185, 214, 215). Tetracycline is contraindicated in pregnant or breastfeeding women and in pediatric patients under 8 years of age due to its toxicity to bone and teeth development, hepatotoxicity, nephrotoxicity, and teratogenicity (185, 216).

Sarecycline, a third-generation narrow-spectrum tetracycline, received FDA approval in 2018 and is the sole tetracycline-class medication specifically indicated for acne therapy (217). Sarecycline, in contrast to conventional first- and second-generation tetracyclines, has a diminished effect on the gut microbiome, hence minimizing gut microbiota disturbance and related adverse effects (217). Furthermore, sarecycline is the only orally administered antibiotic indicated for acne treatment with a label stating low resistance potential (214).

The inappropriate use and misuse of antibiotics have elevated the issue of antimicrobial resistance to one of the most serious challenges facing global public health in the 21st century (218, 219). It is estimated that in 2019, 4.95 million deaths worldwide were directly or indirectly attributable to antimicrobial resistance; if strong countermeasures are not taken, this number could rise to 10 million annually by 2050 (220). In dermatology, acne is the most common skin disease worldwide. C. acnes plays a key role in the pathogenesis of acne, and antibiotics are frequently recommended for its treatment, leading to the emergence of C. acnes strains resistant to antibiotics globally (213). The guidelines note that in 2021, dermatologists prescribed more oral antibiotics per clinician than any other specialty, with the majority of these antibiotics used to treat acne. The guidelines note that in 2021, dermatologists prescribed more oral antibiotics per clinician than any other specialty, and the majority of these antibiotics were used to treat acne (185). Furthermore, in a U.S. study of 29,908 patients treated with oral antibiotics for acne, 64% received treatment for more than 3 months, and 17% of them were treated for more than 6 months (216). It is the overuse of topical over-the-counter antibiotics in some countries that have led to the global emergence of C. acnes antibiotic-resistant strains. Currently, a significant proportion of C. acnes strains have developed resistance to commonly used antibiotics (clindamycin, erythromycin), and in some countries, the resistance rate to clindamycin and erythromycin can reach 70%–90% (213). Although the current resistance rate of C. acnes to tetracyclines is low (typically <5%, with some European studies reporting 0%), there is an upward trend (213). The current severe situation regarding antibiotic resistance informs the latest international guidelines for acne treatment. The guidelines recommend: First, limiting the duration of systemic antibiotic use to no more than 3–4 months; Second, systemic antibiotics must not be used alone but must be combined with BPO or topical retinoids to reduce the risk of resistance; Third, topical antibiotics are permitted only in the form of fixed-dose combinations containing BPO; Fourth, prioritize non-antibiotic regimens, such as BPO, topical retinoids, combined oral contraceptives (COCs) or spironolactone for women, and clascoterone (185, 221–223).

#### 6.1.6 Hormone therapy

Hormonal treatment for acne refers to regulating sebaceous gland activity by modulating androgen levels. This treatment approach is particularly suitable for the following conditions: first, sex hormone-related acne (such as acne associated with polycystic ovary syndrome or linked to the menstrual cycle); and second, moderate-to-severe acne in women presenting with signs of hyperandrogenism (hirsutism, hair loss, and menstrual irregularities) (224). Currently, commonly used medications include several categories: first, COCs; second, spironolactone; and third, newer topical antiandrogens (such as clascoterone) (185, 224).

Oral COCs are the main medications for acne hormone therapy, consisting of estrogen and progestin. Estrogens, including ethinyl estradiol, diminish free androgens by inhibiting ovarian androgen secretion via suppression of the hypothalamic-pituitary-gonadal axis and promoting hepatic synthesis of sex hormone-binding globulin, which subsequently reduces sebum production (185, 224). Certain progestins, such as drospirenone and norethisterone, structurally resemble spironolactone and can directly antagonize androgen receptors on the epidermis (224). Moreover, the downregulation of free androgen levels by COCs diminishes sebum secretion, indirectly inhibiting the anaerobic conditions necessary for C. acnes life. This suppresses the proliferation of C. acnes and the inflammatory response triggered by this bacterium (224).

Spironolactone is a potassium-sparing diuretic that diminishes androgen levels and activity by competitively antagonizing androgen receptors and inhibiting 5-α reductase, thereby downregulating sebum production (185, 198). Previous concerns about hyperkalemia caused by this medicine required electrolyte surveillance throughout the treatment. Molly Plovanich conducted a retrospective investigation revealing that the incidence of high serum potassium levels in healthy young women taking spironolactone for acne therapy did not significantly differ from the natural occurrence rate in the general healthy population. Consequently, monitoring serum potassium levels is unnecessary (225).

Compared with alternative medications, oral COCs and spironolactone are more appropriate for the long-term treatment of acne. Management guidelines for acne suggest that within three months, oral COCs are less effective than oral antibiotics are; however, by six months, the efficacy of both treatment modalities becomes comparable (185). Consequently, hormone therapy for acne may act as a substitute for antibiotics (72, 226). Importantly, both medications have significant adverse effects. Oral COCs may induce breast soreness, mood alterations, gastrointestinal disturbances, weight gain, headaches and breakthrough bleeding and may also increase the risk of venous thromboembolism, myocardial infarction, stroke, breast cancer, and cervical cancer. Spironolactone may lead to gynecomastia and feminization of male fetuses (72, 185, 198). Therefore, the administration of these two categories of medications is prohibited in male patients and some female patients.

However, this difficulty has been efficiently handled with the successful creation of the new topical antiandrogen medication Clastocortron (227). Clascoterone is the first FDA-approved topical anti-androgen that competitively binds to androgen receptors in sebaceous glands, thus obstructing the acne-inducing actions of dihydrotestosterone (228, 229). This medication inhibits the production of pro-inflammatory cytokines in a dose-dependent manner (196, 197). Compared with traditional anti-androgens, the distinctive molecular structure of clascoterone enables high topical skin accumulation. Adverse effects are confined to moderate erythema and desquamation/dryness at the site of application, with incidence rates comparable to those of control vehicle or placebo creams (228, 230). No systemic adverse effects were detected in the clinical setting (228).

#### 6.1.7 Physical modalities

Physical therapy for acne typically refers to non-pharmaceutical physical interventions that directly target the skin to improve the pathophysiological processes of acne. This therapy encompasses acne lesion extraction, chemical peels, laser and light therapy, microneedle radiofrequency, and photodynamic therapy (185, 231). Physical treatment directly targets localized skin lesions and addresses the pathophysiology of acne, with notable advantages: precise targeting, substantial efficacy, elevated safety (absence of systemic side effects, only temporary local skin reactions), and high patient adherence. It is especially appropriate for patients who are intolerant to or unresponsive to conventional pharmacotherapy, those exhibiting antibiotic resistance, those experiencing acne during pregnancy, or those needing prolonged maintenance medication (231).

Conventional physical therapies, such as comedone extraction and cryotherapy with a carbon dioxide slurry, the former removes skin debris from hair follicles and enhances localized comedone dissolution; the latter is an effective cryotherapy treatment for acne. Although effective, modern approaches favor treatments such as laser and light therapy (231). Among these, 5-aminolevulinic acid photodynamic therapy (ALA-PDT) has gradually demonstrated advantages in acne management due to its unique mechanism of action, minimally invasive nature, absence of systemic side effects, and clear clinical efficacy (72, 185). Its mechanism of action is as follows: ALA-PDT stimulates the COX2/PGE2 axis in Keratinocytes, promoting PGE2 secretion. PGE2 subsequently acts on the TLR4 on the surface of macrophages, enhancing the expression of TREM1 in macrophages. This process activates the p38 MAPK and NF-κB signaling pathways, promoting M1 polarization and enhancing the acute inflammatory response, thereby interrupting the chronic inflammatory cycle of acne (80). This mechanism is entirely opposite to traditional anti-inflammatory treatments that promote M2 macrophages polarization. Additionally, the photosensitizer ALA generates singlet oxygen upon red light stimulation, which is effective in eliminating C. acnes (80). It is worth noting that PDT treatment often leads to severe local skin inflammation, typically characterized by erythema and pustules. The inflammatory reaction reaches its peak approximately 4 hours after treatment and returns to baseline levels within roughly 24 hours (232, 233). Furthermore, research suggests that the treatment efficacy of ALA-PDT is positively correlated with the intensity of the acute inflammatory response following treatment (80, 185, 233).

#### 6.1.8 Biological agent

With the continuous exploration of the pathophysiological mechanisms and inflammatory pathways of acne, relevant research has laid a theoretical basis for the development of new treatment strategies. Inflammatory cytokines, including IL-1β, IL-17, IL-23, and TNF-α, have been shown to be closely associated to the inflammatory responses in acne. Thus, biologics that target TNF-α, IL-17, IL-23, and IL-1β may promise the treatment of acne (72, 74). For example, adalimumab, which targets TNF-α, has shown off-label effectiveness in treating acne conglobata, resulting in clinical improvement and no recurrence of lesions (234, 235). Furthermore, secukinumab (236) and risatumumab (237). Moreover, secukinumab (236) and risatumumab (237), which target IL-17 and IL-23, have been documented to mitigate SAPHO syndrome, a rare chronic inflammatory illness characterized by synovitis, acne, pustulosis, osteosclerosis, and osteitis. Currently, the canakinumab targeting IL-1β has demonstrated therapeutic effects in various inflammatory diseases such as psoriasis, rheumatoid arthritis, and gout (238, 239). Its mechanism of action is to neutralize the excessive activity of IL-1β (238–240). Although there are no reports on the use of antibodies targeting IL-1β for the treatment of acne vulgaris at present, only a few studies have mentioned the application of canakinumab in severe acne related to rare genetic diseases such as PAPA syndrome (241, 242). However, given the central role of IL-1β in the inflammatory response of acne (104, 243), although the clinical evidence is not yet sufficient, this type of biological agent still has certain theoretical feasibility in the treatment of acne.

In addition to targeting inflammatory mediators associated with acne, other innovative.

Biologics agents can exert therapeutic benefits by influencing other pathophysiological mechanisms of acne, including sebum production, follicular keratinization, skin microbiome, and wound healing. Research has demonstrated that IGF-1 facilitates sebocyte proliferation, differentiation, and abnormal follicular keratinization (194). Reports indicate that metformin can alleviate acne symptoms by lowering IGF-1 levels (244), suggesting that biologic antibodies that target IGF-1, such as teprotumumab, could serve as a prospective method for the treatment of acne (74). Additionally, novel biotherapies targeting the skin microbiota or C. acnes, such as probiotics (245), antimicrobial peptides (246), and bacteriophages (247), have demonstrated significant potential in acne treatment. Antimicrobial peptides and phage treatment exhibit direct antibacterial or bactericidal capabilities, directly targeting pathogenic bacteria while circumventing the emergence of drug resistance (246, 247). Unfortunately, no clinical studies have reported the therapeutic efficacy of these two biological therapies. Additionally, Teruaki Nakatsuji reported that administering anti-CAMP factor serum to mice in an acne model markedly diminished C. acnes-induced macrophage and keratinocyte mortality while mitigating acne-related inflammation, thereby establishing a theoretical foundation for the development of a CAMP factor vaccine (248). Liu et al. similarly illustrated the therapeutic potential of CAMP factor-targeting vaccinations via animal studies (249). Regrettably, no research has yet assessed the effectiveness of these vaccinations in humans.

Biological treatments for acne exhibit considerable promise for future acne care owing to their precise targeting, few adverse effects, and efficacy in addressing resistant cases. However, biologic therapy also has many limitations. First, there is a lack of large-scale clinical trials, so its efficacy and safety require further evaluation; second, there is variability among individuals; third, treatment costs are relatively high; and fourth, they may even lead to immunosuppression (250, 251). Taken together, these factors will limit the widespread clinical adoption of biologics, indicating that there is still a long way to go before they are broadly applied in clinical practice (**Table 1**).

**Table 1:** This table shows the standard clinical treatments for acne, including treatment examples, importance, mechanism of action, common side effects, solutions for side effects.

Treatment examples		Importance	Mechanism of action	Common side effects	Solution for side effects	References
retinoids	Topical retinoids(ATRA, adapalene, tigfarlotin)	First-line treatment; the cornerstone of acne management.	improves abnormal keratinization of hair follicle epithelium; anti-inflammatory; regulates lipid metabolism.	Long treatment duration; skin irritation reactions.	Certain skin cosmetics	(185–187, 190, 252)
	Oral retinoids (Isotretinoin)	First-line treatment	Potently inhibits sebum secretion; normalizes follicular keratinization; anti-inflammatory; indirectly antibacterial.	Dry skin, teratogenicity, cheilitis, epistaxis, skin sensitivity, abnormal liver function, reduced ovarian reserve, risk of depression, and hyperlipidemia.	Side effects are dose-dependent and can be effectively prevented by controlling the dosage.	(74, 185, 191–196)
BPO		First-line treatment	Antibacterial; anti-inflammatory; dissolves acne.	Burning sensation, stinging, dryness, erythema, pain, peeling, irritation, fabric staining and bleaching, and rare cases of contact allergy.	Microencapsulate BPO gel-sol to delay release rate and prevent initial high-concentration drug burst release; Side effects are dose dependent, reducing concentration.	(41, 72, 197–204)
Azelaic acid		Second-line treatment	Antibacterial; normal keratinization process; anti-inflammatory; regulation of lipid metabolism.	Hypopigmentation	For acne patients with darker skin tones	(185, 205–208)
Antibiotics	Topical (such as clindamycin)	First-line treatment for mild acne; Second-line treatment for moderate to severe acne.	Antibacterial; anti-inflammatory	Drug resistance	In combination with BPO	(185, 210–213)
	Oral (such as tetracyclines)	Second-line treatment for moderate to severe acne	Antibacterial; anti-inflammatory.	Drug resistance, dental and skeletal toxicity, hepatic and renal toxicity, teratogenicity, inflammatory bowel disease, induced lupus erythematosus, idiopathic intracranial hypertension, hyperpigmentation, onycholysis, dizziness, and vertigo.	Sarecycline, a third-generation narrow-spectrum tetracycline, targets C. acnes with low resistance; used in combination with BPO.	(185, 209, 214–217)
Hormone therapy	COCs	Indicated for female patients with hormone-related acne and moderate to severe acne accompanied by hyperandrogenism.	Reduce sebum secretion;Indirect antibacterial effects.	Breast tenderness, gastrointestinal issues, weight gain, and even venous thromboembolism, myocardial infarction, breast cancer, cervical cancer, and other conditions.	Clastocortron	(72, 185, 224, 225, 227–230)
	Spironolactone	—	Competitively antagonizes androgen receptors; inhibits 5-alpha reductase.	Gynecomastia, male fetal feminization.		
	Clastocortron	The first FDA-approved topical anti-androgen medication.	Competitively binds to androgen receptors in sebaceous glands; inhibits the acne-promoting effects of dihydrotestosterone.	None	—	
Physical mode (ALA-PDT)		Precise targeting, significant therapeutic efficacy, high safety profile, no systemic side effects, and high patient compliance.	ALA-PDT promotes M1 polarization through the COX2/PGE2 axis, enhancing acute inflammatory responses and disrupting the chronic inflammatory cycle of acne.	Severe inflammatory reaction in localized skin areas.	Therapeutic efficacy of ALA-PDT correlates positively with the intensity of the local acute inflammatory response.	(80, 185, 231, 232)
Biological agent	Adalimumab, secukinumab and rituximab	Precision targeting with minimal side effects, suitable for treatment-resistant acne.	Targeted inhibition of TNF-α, IL-17 and IL-23	Lack of large-scale clinical studies, efficacy and safety remain to be evaluated, significant individual variability, high cost, and even immunosuppression.	—	(72, 74, 234–237)
	Tepudumab	—	Targeted inhibition of IGF-1	No clinical trial evidence is currently available.	—	(74, 194, 244)
	Antimicrobial Peptides and bacteriophages	Does not produce drug resistance.	Antimicrobial (antimicrobial peptides) or bactericidal (bacteriophages).	—	—	(246, 247)

### 6.2 Innovative therapeutic approaches based on macrophage polarization

Auricular acupuncture, a prominent therapeutic method in traditional Chinese medicine(TCM), produces therapeutic effects by stimulating specific points on the outer ear. This method exhibits distinct anti-inflammatory properties (253). Zhuo et al. conducted auricular acupuncture therapies in an acne rat model, comprising auricular bloodletting therapy (ABT), auricular puncture (APS), and their combination (ABPS). The results indicated that all three methods—ABT, APS, and ABPS—mitigated acne inflammation by downregulating M1 and upregulating M2, thus decreasing serum TNF-α and IL-1β levels (254). Furthermore, APS was found to potentially regulate macrophage polarization by inhibiting TLR2/NF-κB signaling. This work presents initial evidence that auricular therapy alleviates acne inflammation through the modulation of macrophage polarization. While the pathophysiology and pathological mechanisms of human acne are more intricate than those of in rat, this research enriches the theoretical basis of TCM auricular therapy for acne treatment and supports its future clinical application in acne treatment.

**Figure 5:**
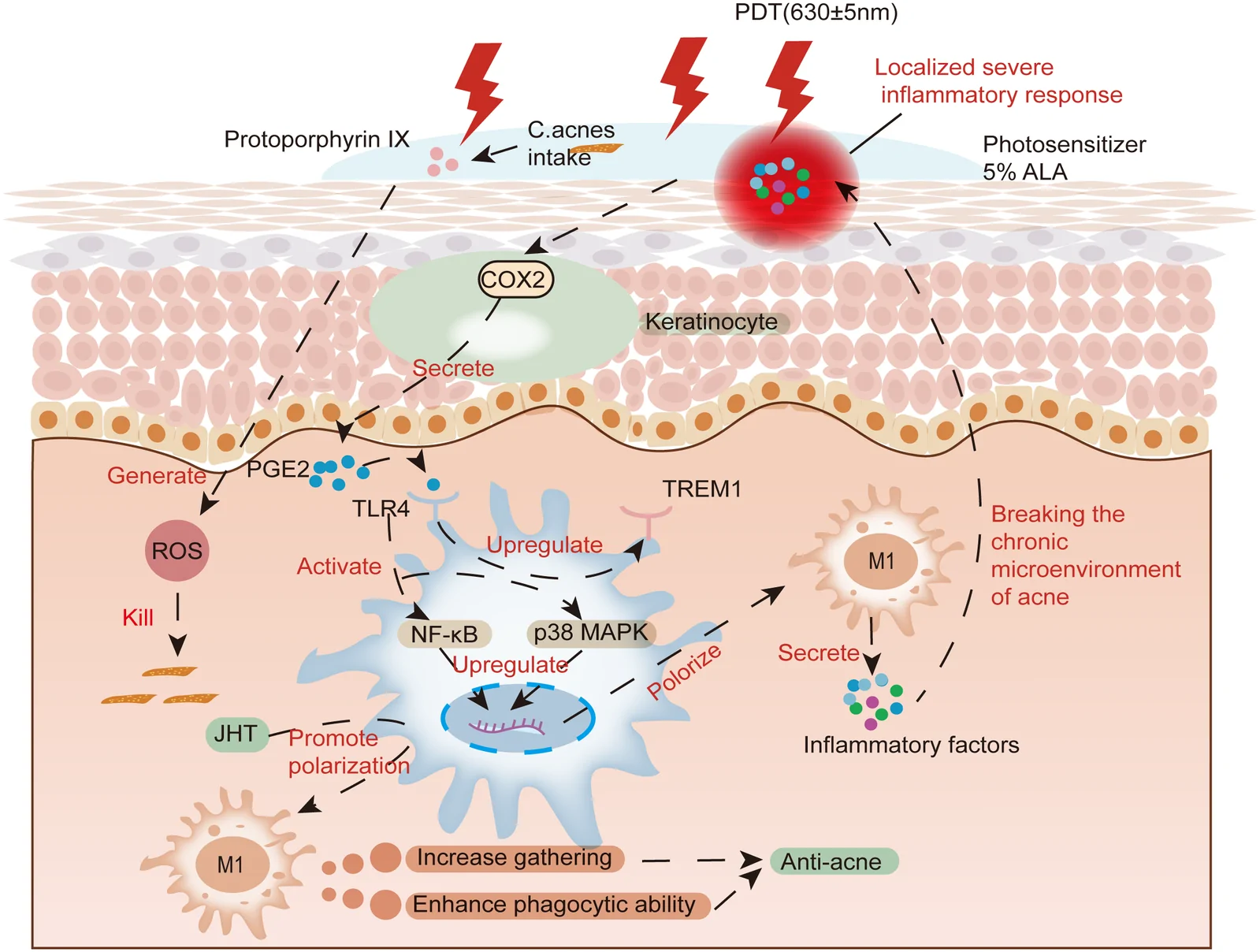
This figure shows the unique therapeutic mechanisms of ALA-PDT and JHT. Both JHT and ALA-PDT achieve therapeutic effects through the promotion of M1 macrophage polarization: the former mitigates C. acnes-induced inflammation by augmenting macrophage functionality, whereas the latter interrupts the chronic inflammatory cycle of acne by amplifying the acute inflammatory response.

Moreover, several traditional Chinese medicines or natural compounds exert anti-acne effects by modulating macrophage polarization (14, 76, 102, 255, 256). For instance, Tang et al. reported above identified that sebaceous glands facilitate the upregulation of M1 in acne inflammatory situations. CBD mitigates acne by reversing the polarization process (105). Research has indicated that baicalin, a flavonoid derived from Scutellaria baicalensis, markedly decreases the expression of the M1 macrophage-associated cytokines IL-1β, IL-6, IL-8, TNF-α, and NLRP3 in a rat acne model (257). Although this work did not directly mention alterations in M1/M2 surface indicators, research has demonstrated that luteolin (another flavonoid compound) mitigates inflammation in acute lung injury by enhancing M2 polarization via the upregulation of IL-10 expression (258). Consequently, we can deduce that the anti-acne mechanism of baicalin could involve the transformation of macrophage phenotypes. Similarly, the natural flavonoid quercetin exhibits anti-acne properties by facilitating M2 macrophage polarization (76). Furthermore, Zhao et al. speculated that many natural plant products—including schisandrin, glycyrrhetinic acid, diterpenoids, *Artemisia annua*, coriandrol, and Jumihaidokuto—may have anti-acne properties through the modulation of macrophage polarization (76). Among these, research has indicated that JHT, a conventional Japanese acne treatment, mitigates C. acnes-induced inflammation by facilitating M1 macrophage polarization, augmenting macrophage aggregation, and increasing phagocytic efficacy (259). Both JHT and ALA-PDT achieve therapeutic effects through the promotion of M1 macrophage polarization: the former mitigates C. acnes-induced inflammation by augmenting macrophage functionality, whereas the latter interrupts the chronic inflammatory cycle of acne by amplifying the acute inflammatory response. (**Figure 5**) Their distinctive mechanism of increasing M1 distinguishes them from traditional anti-inflammatory methods that support M2 polarization, providing a novel therapeutic approach for acne.

Furthermore, in the pathological process of acne, C. acnes acts on TLR2/4 on the surface of macrophages, promoting their polarization toward the M1 type, whereas IL-4 and IL-10 induce M2 polarization (259). On the basis of this mechanism, future strategies may consider combining TLR antagonists with M2 polarization inducers to modulate the M1/M2 ratio, thereby exerting anti-acne effects.

### 6.3 Potential therapies for the transplantation of MSCs and their derivatives

MSCs and their derivatives have consistently been a central focus in regenerative medicine research owing to their remarkable biological characteristics, especially their immuno-inflammatory regulating capabilities. Since Hillard Lazarus initially examined MSCs as a cellular therapy in humans in 1995 (260), over three decades of advancements have resulted in numerous MSC-based clinical studies globally, significantly exceeding the research size of other cell types (261). Although the therapeutic efficacy of MSCs has been proven in multiple animal disease models, the majority of clinical trials involving MSCs have not yielded the anticipated results due to several issues. These factors include heterogeneity, immunocompatibility, stability, cell viability, administration methods, host factors, dosage discrepancies, and diminished homing rates (120, 262). The therapeutic efficacy of MSCs in facilitating M2 polarization has been corroborated in many animal disease models, encompassing but not limited to wound healing (263, 264), lung disease (265), heart disease (266), kidney disease (267), spinal cord injury (268), and brain injury (269). The MSC-based drug Prochymal was initially conditionally approved in Canada, New Zealand, and Japan for the treatment of pediatric graft-versus-host disease. Nonetheless, its clinical implementation has encountered obstacles owing to reimbursement issues. Encouragingly, in 2018, the European Commission authorized Alofisel^®^, the first MSC-based medication, for the treatment of extraintestinal fistulae associated with Crohn’s disease (261). Additionally, MSC-based therapeutics have gained approval for the treatment of subcutaneous tissue injury, acute myocardial infarction, degenerative osteoarthritis, amyotrophic lateral sclerosis, limb ischemia, vasculitis, and autoimmune diseases, including rheumatoid arthritis, systemic lupus erythematosus, and multiple sclerosis (270, 271). Although the range of diseases currently approved for MSC drug applications is far narrower than that demonstrated in animal studies, this situation is improving with the continuous advancement and expansion of clinical research, indicating that MSC therapeutic strategies hold broad clinical application prospects.

Currently, investigations into the application of MSCs and their derivatives for acne treatment remain in the initial exploratory stage, with the literature predominantly addressing the management of post-acne scarring. Research has demonstrated that adipose-derived stem cells (ADSCs) have multiple therapeutic effects on rabbit ear acne models, including anti-inflammatory properties, enhancement of collagen remodeling, and promotion of scar epidermal normalization, thus significantly improving post-acne scarring. Moreover, the concomitant administration of ASC-conditioned medium amplifies the therapeutic effects (272). Several clinical trials have proven the effectiveness of MSCs in treating acne scars. Zeinab enrolled 14 patients with moderate to severe acne scars and delivered intradermal injections of autologous bone marrow-derived stem cells into each scar. After six months, all patients exhibited improvement in acne scars without any side effects (273). Furthermore, Rania S Abou Eitta revealed that a single autologous ASC transplant produced effects comparable to three sessions of fractional carbon dioxide laser therapy in ten patients with post-acne scarring (274). El-Domyati similarly reported that the combination of AF-MSC-CM and microneedling was more effective than microneedling alone in the treatment of atrophic acne scars (275). These studies demonstrate the therapeutic efficacy of MSCs for acne scars. The limited sample sizes in these studies require more clinical trials to assess the efficacy and safety of MSCs and their derivatives.

In addition to acne scars, the therapeutic potential of MSCs in acne treatment has been reported. Li et al. discovered that ADSCs mitigate acne inflammation by inhibiting NLRP3 inflammasome activation, which relies reliant on mitochondrial ROS, consequently decreasing IL-1β secretion (243). Research indicates that berberine facilitates M2 polarization of macrophages by obstructing NLRP3 inflammasome activation-induced M1 polarization, thus exerting significant neuroprotective effects and mitigating inflammation after peripheral nerve injury (276). This study indirectly emphasized the crucial function of NLRP3 in facilitating M1 macrophage polarization. Consequently, although Li’s research did not explicitly involve macrophage polarization, ADSCs exert anti-inflammatory effects by blocking the NLRP3/IL-1β pathway, likely involves the conversion of M1 to M2 macrophages. This hypothesis requires further validation through the analysis of surface markers on M1 or M2 macrophages using flow cytometry or qPCR. Zhao et al. found that injecting a gel containing autologous subcutaneous vascular matrix components and stem cells into the facial area effectively alleviates inflammation caused by C. acnes (277). Yu et al. found that C. acnes can induce the formation of neutrophil extracellular traps (NETs). ADSCs can inhibit this process by activating the Nrf2 signaling pathway, thereby suppressing the abnormal proliferation of Keratinocytes and exerting anti-inflammatory effects (278). In addition, Sein Hwang and his team reported that hUCB-MSCs pre-treated with TLR3 and TLR4 can markedly enhance their anti-inflammatory and immunomodulatory properties via EVs. This study also showed that EVs alone possess effectiveness comparable to intact MSCs, showing that EVs are essential carriers for MSC-mediated immunoregulation. This establishes a theoretical foundation for employing TLR-pretreated MSC-EVs as an acellular therapeutic, avoiding possible hazards linked to stem cell treatment (such as tumorigenicity) while facilitating transport and storage capabilities (279).

Although MSC transplantation therapy for acne currently has a certain theoretical basis and is supported by animal study data, numerous challenges remain to be overcome before it can be truly implemented in clinical practice. For example, differences in cellular heterogeneity and immunogenicity resulting from MSCs derived from different tissue sources; the lack of standardized preparation and expansion protocols; low survival rates, low directed homing rates, and low retention rates following *in vivo* transplantation; diverse administration routes but no established optimal protocol; varying dosages but no universally recognized optimal effective dose; tumorigenic risk; and ethical controversies, among others (116, 120, 262, 280). Although MSC-Exos are often regarded as a “cell-free therapeutic strategy” that can replace whole MSCs, with comparable efficacy to whole MSCs and possessing advantages such as no tumorigenic risk, lower immunogenicity, fewer ethical controversies, and better stability, they also face numerous challenges (134, 281). It is clear, therefore, that achieving the safe, effective, and reproducible clinical translation of MSCs will require extensive basic research and clinical trials to address each of the aforementioned bottlenecks and limitations.

## 7 Conclusions and outlook

In recent years, with the continuous advancement of acne research, a series of innovative therapies have emerged to overcome the limitations of traditional treatment regimens. These include tetracycline derivatives such as sarecycline; antiandrogens such as spironolactone; and topical androgen receptor antagonists such as clascoterone, biologics, and traditional Chinese medicinal therapies. While the etiological mechanisms of acne remain unclear, existing research has clearly demonstrated that macrophages play a pivotal regulatory role in the disease course. Acne is essentially an inflammatory skin condition. Therefore, anti-inflammatory strategies that promote M2 macrophage polarization through the transplantation of MSCs or their derivatives are supported by theoretical evidence. Moreover, as investigations into MSCs and macrophages deepen, increasing evidence endorses macrophage polarization as a potential therapeutic path. Nonetheless, investigations into the therapeutic efficacy of MSCs and their derivatives in modulating macrophages for acne treatment are scarce, with the majority of research concentrating on acne scar repair. Among the few studies on acne, only the inhibition of pro-inflammatory factor expression by MSCs has been reported, with no direct evidence of macrophage phenotypic transformation. Therefore, we can only indirectly speculate that its anti-inflammatory effect is achieved by promoting polarization of M2 macrophages. Furthermore, these results predominantly stem from animal experiments or preclinical research, which lack confirmation of the effectiveness and safety of the therapy through extensive, long-term clinical trials. In addition to verifying its effectiveness and safety, future research should also focus on various challenges that MSCs may encounter during the clinical translation process and address them one by one. For example, attempting to use MSCs derived from induced pluripotent stem cells for animal and clinical trials to avoid the heterogeneity and ethical disputes brought by MSCs from different tissue sources; continuously optimizing the preparation process of MSCs to promote the establishment of standardized preparation and expansion procedures; optimizing the delivery methods of MSCs (hydrogel, scaffold, microspheres, etc.), improving the survival rate, directed homing ability, and retention rate of MSCs after transplantation; advancing large-scale preclinical and clinical trials, and striving to clarify the optimal drug delivery route and effective dose range, etc.

The theoretical basis for the MSCs and their derivative transplantation therapies discussed in this review lies in the fact that acne is essentially an inflammatory disease. MSCs and their derivatives exert an anti-inflammatory effect by promoting the polarization of M2 macrophages, thereby achieving the goal of treating acne. However, as research on macrophages continues to deepen, existing studies indicate that the roles of M1 and M2 macrophages at various stages of acne are not strictly harmful or beneficial. “Harmful” M1 macrophages also eliminate C. acnes in the early stages of acne. Conversely, excessive activation of “beneficial” M2 macrophages can lead to scar hyperplasia in the later stages. Based on this, an ideal MSCs treatment strategy should possess stage-specific, precise regulatory capabilities: by transplanting MSCs and their derivatives, M1 macrophages should be moderately activated in the early stages of acne to clear C. acnes, while avoiding excessive activation that could cause tissue damage. In the middle stages, after the bacterial load has decreased, the conversion of M1 to M2 should be promoted to limit the spread of inflammation. In the late stage, sustained M2 activation must be suppressed to reduce the risk of scar formation. To achieve the goal of precise, stage-specific regulation described above, various technologies such as single-cell sequencing, spatial transcriptomics, and *in vivo* imaging techniques will need to be combined to conduct in-depth research on the precise spatiotemporal patterns of macrophages in the acne disease process in future.

Despite the nascent stage of research on MSCs and their derivatives for acne treatment, characterized by a scarcity of foundational studies and clinical data, the rapid advancement of regenerative medicine and the increasing demand for acne therapies may promote the emergence of a large amount of basic and clinical research. These studies will enable in-depth exploration of the efficacy, safety, and feasibility of MSC therapies for treating acne.
